# Cyclooxygenase-2/Prostaglandin E2 Pathway Facilitates Infectious Bronchitis Virus-Induced Necroptosis in Chicken Macrophages, a Caspase-Independent Cell Death

**DOI:** 10.3390/v17040503

**Published:** 2025-03-31

**Authors:** Motamed Elsayed Mahmoud, Dylan Tingley, Akeel Faizal, Awais Ghaffar, Muhammed Azhar, Doaa Salman, Ishara M. Isham, Mohamed Faizal Abdul-Careem

**Affiliations:** 1 Faculty of Veterinary Medicine, University of Calgary, 3330 Hospital Drive NW, Calgary, AB T2N 4N1, Canada; motamed.ali@ucalgary.ca (M.E.M.); akeel.faizal@ucalgary.ca (A.F.); awais.ghaffar@ucalgary.ca (A.G.); muhammad.azhar1@ucalgary.ca (M.A.); doaa.salman@ucalgary.ca (D.S.); fathimaishara.muhamm@ucalgary.ca (I.M.I.); 2 Department of Animal Husbandry, Faculty of Veterinary Medicine, Sohag University, Sohag 84524, Egypt

**Keywords:** infectious bronchitis virus, COX-2/PGE2 pathway, caspases, pyroptosis, necroptosis, NLRP3 inflammasome, RIPK1

## Abstract

Infectious bronchitis virus (IBV) poses a major challenge to poultry health and productivity. This study examined how inflammatory cell death pathways influence the replication and pathogenesis of two IBV strains—respiratory Connecticut (Conn) A5968 and nephropathogenic Delmarva (DMV)/1639—in chicken macrophages. Low serum conditions enhanced viral replication, reduced cell viability, and promoted apoptosis and necroptosis, with DMV/1639 showing more pronounced effects. Modulation of the cyclooxygenase-2/prostaglandin E2 (COX-2/PGE2) pathway displayed strain-specific effects, mitigating necroptosis in DMV/1639-infected cells but exacerbating apoptosis and necroptosis in Conn A5968-infected cells. Broad caspase inhibition (z-VAD-FMK) reduced necroptosis, while selective caspase-1/4 inhibition heightened apoptotic responses. Caspase-8 inhibition selectively reduced necroptosis in DMV/1639 infections but increased apoptosis and necroptosis in Conn A5968 infections. NLRP3 inflammasome and RIPK1 inhibition decreased cell viability and increased apoptosis in both strains but had distinct effects on necroptosis. These findings reveal the strain-specific regulation of viral replication, apoptosis, and necroptosis, underscoring the intricate interplay between IBV and host inflammatory pathways. Understanding these mechanisms provides novel insights into IBV pathogenesis and highlights potential therapeutic strategies to mitigate its impact on poultry health.

## 1. Introduction

Infectious bronchitis virus (IBV), a member of the Coronaviridae family, is a major pathogen of poultry, first identified in the early 1930s, decades before the discovery of human coronaviruses [1]. It creates significant challenges for the poultry industry, targeting the respiratory, renal, and reproductive systems, leading to substantial economic losses [2]. Canadian IBV variants, such as Delmarva (DMV)/1639 and Connecticut (Conn) A5968, exhibit distinct pathogenicity, with DMV/1639 being nephropathogenic and Conn A5968 primarily affecting the respiratory system [3,4]. Despite advances in understanding IBV pathogenesis, critical gaps remain in the interplay between IBV and host immune responses, particularly in macrophages, which serve as key sentinels in innate immunity [5].

Programmed cell death (PCD), including apoptosis, pyroptosis, and necroptosis, plays a vital role in controlling viral infections [6]. Apoptosis ensures the elimination of virus-infected cells in a controlled manner [7], while necroptosis, a form of regulated necrosis, acts as a backup when apoptosis is inhibited [8]. These pathways are crucial in macrophages, where PCD determines the outcome of viral infections and shapes immune responses [5]. However, the dual roles of these pathways in facilitating viral clearance versus enhancing pathogenesis remain poorly understood. Recent studies have highlighted controversial findings, particularly in how specific vaccinal viral strains modulate these pathways to their advantage [9], underscoring the need for further investigation.

The cyclooxygenase-2 (COX-2)/prostaglandin E2 (PGE2) pathway has emerged as a critical regulator in viral infections, influencing macrophage survival, apoptosis, and necroptosis [10]. While COX-2/PGE2 activation has been implicated in facilitating IBV replication in macrophages [11], its precise role in modulating PCD remains ambiguous. The previously reported dual role, where PGE2 can either promote apoptosis or enhance macrophage survival [12], showcases its involvement in host–pathogen interactions and its potential as a therapeutic target.

Caspases, NOD-like receptor family pyrin domain containing three (NLRP3) inflammasomes, and Receptor-Interacting Protein kinases (RIPKs) are essential regulators of PCD, playing key roles in immune responses during viral infections. Caspases, particularly caspase-8, are crucial mediators of apoptosis and necroptosis, functioning as initiators and executioners of cell death pathways. Caspase-8 has been shown to regulate both apoptotic and necroptotic pathways, with its inhibition leading to shifts between these forms of cell death, depending on the context [13]. Similar to mammals, the NLRP3 inflammasome in chicken macrophages is activated by pathogen-associated molecular patterns (PAMPs) and danger-associated molecular patterns (DAMPs), triggering the secretion of pro-inflammatory cytokines interleukin (IL)-1β and IL-18 [14]. NLRP3 activation has been linked to both apoptosis and pyroptosis, and its inhibition can alter immune responses and cell survival, suggesting a complex role in viral infections [15], including IBV [16]. RIP kinases, particularly RIPK1 and RIPK3, mediate necroptosis, a programmed form of necrosis. RIPK1 plays a central role in coordinating cell survival and death decisions by integrating signals from both apoptosis and necroptosis pathways [17]. The interplay between these molecules in regulating viral replication and host immune responses remains an area of active investigation.

This study aims to elucidate the role of the COX-2/PGE2 pathway in regulating programmed cell death during IBV infection in chicken macrophages, focusing on its impact on apoptosis and necroptosis. Furthermore, we investigated the effects of caspase inhibitors, as well as the inhibition of the NLRP3 inflammasome and RIPK1, to dissect their contributions to viral replication and cell death mechanisms. By exploring the interplay between these pathways, this research provides critical insights into IBV pathogenesis and identifies potential targets for mitigating its impact on poultry health.

## 2. Materials and Methods

### 2.1. Viruses

Two IBV strains, Canadian DMV/1639 (DMV/1639) and Connecticut (Conn A5968), were used in this study. The DMV/1639 strain underwent molecular characterization and titration as has been previously described [11]. The Conn A5968 strain, obtained from the American Type Culture Collection (ATCC, Manassas, VA, USA), has been extensively utilized in prior research [18].

### 2.2. Chicken Macrophage Cell Line

The MQ-NCSU cell line is a chicken mononuclear phagocyte cell line developed at North Carolina State University (NCSU) and was provided by Dr. Shayan Sharif (University of Guelph, ON, Canada) and was cultured following standard protocols [19]. Cells were maintained in LM-Hahn medium supplemented with 1% antibiotic/antimycotic and 10 µM 2-mercaptoethanol. After 24 h in complete media, the cells were transferred to culture media containing varying concentrations of fetal bovine serum (FBS; 0%, 2%, 4%, and 8%) or chicken serum (0%, 2.5%, 5%, and 10%). All culture reagents were procured from Gibco Life Technologies (Burlington, ON, Canada).

### 2.3. Reagents and Treatment

The selective COX-2 inhibitor SC-236, prostaglandin PGE2, and caspase inhibitors (caspase-1/4 VX765, caspase-3, and caspase-9) were obtained from Millipore Sigma (Burlington, MA, USA). The caspase 8 (z-EID-FMK) and pan-caspase inhibitor z-VAD-FMK were sourced from MedChemExpress (Monmouth Junction, NJ, USA). Bromosulfophthalein sodium (BSP), a prostaglandin release inhibitor, was dissolved in phosphate-buffered saline (PBS), while other compounds were dissolved in dimethyl sulfoxide (DMSO; Thermo Fisher Scientific, Rockford, IL, USA). Necrostatin-1, MCC950, and Necrostatin were used for NLRP3 inflammasome and RIPK1 inhibition, respectively. Non-cytotoxic and effective doses of the COX-2 inhibitor SC-236, PGE2, and the EP receptor blockers TG4-155 and L1-61 were determined based on prior studies [11,18]. IBV-infected chicken macrophages were treated with inhibitors (10 µg/mL) targeting caspase-1/4, caspase-3, caspase-9, or pan-caspase (z-VAD-FMK; 20 µM) for 24 h. Treatments were conducted in serum-reduced culture media (4.5% serum) to evaluate the interaction of the treatments with viral replication and cell death pathways.

### 2.4. Experimental Design

MQ-NCSU cells were seeded in 12-well plates at a density of 1.0 × 10^6^ cells/mL in complete media and incubated at 40 °C with 5% CO_2_ for 24 h. After this period, the media were replaced with serum-reduced media, and the cells were infected with IBV at a multiplicity of infection (MOI) of 0.1. Mock-infected controls received Dulbecco’s phosphate-buffered saline (dPBS; Sigma Aldrich, Burlington, MA, USA). Following a 1-h adsorption period, cells were washed and treated with the specified inhibitors. At 24 h post-infection (hpi), supernatants were collected, and cells were washed three times with dPBS before being lysed in Trizol^®^ reagent (Invitrogen Canada Inc., Burlington, ON, Canada). Both lysates and supernatants were stored at −80 °C for subsequent analyses.

### 2.5. Determination of Intracellular and Extracellular IBV Genome Load by Reverse Transcription and qPCR

RNA from both cellular and culture supernatant (SNF) samples was extracted using Trizol^®^ reagent (Invitrogen Canada Inc., Burlington, ON, Canada) following the manufacturer’s instructions. The RNA concentration was quantified using the Nanodrop 1000 spectrophotometer (Thermo Scientific, Waltham, MA, USA). Complementary DNA (cDNA) was synthesized from 2 µg of cellular RNA and 1 µg of supernatant RNA using the SuperScript IV Reverse Transcriptase kit (Thermo Fisher Scientific, Waltham, MA, USA) according to the manufacturer’s guidelines. Real-time quantitative polymerase chain reaction (qPCR) was performed on a CFX Opus Real-time PCR system (BioRad, Hercules, CA, USA) to quantify both the IBV genome load and mRNA expression levels of immune genes. Reactions were carried out in 96-well plates (VWR, Mississauga, ON, Canada), with each sample analyzed in duplicate. The qPCR protocol consisted of an initial denaturation step at 95 °C for 20 s, followed by 39 cycles of amplification at 95 °C for 3 s and 60 °C for 30 s, with final annealing at 95 °C for 10 s. Primer sequences were selected from previously published sources [11,18]. The SYBR Green method was employed, and a three-segment melt curve analysis was conducted. The viral genome copy number was calculated based on a linear dynamic range of 1–7 log10 copies/reaction.

### 2.6. Apoptosis and Necroptosis Assay

To assess cell viability, early and late apoptosis, as well as necroptosis, we utilized the PE-Annexin-V apoptosis detection kit (BD Pharmingen, San Jose, CA, USA). according to the manufacturer’s instructions (BD Pharmingen, USA). Flow cytometric analysis was performed, and gating analysis was carried out as previously described (Supplementary Figure S1).

### 2.7. Caspase-1 and Caspase-9 Assays

Caspase-1 and caspase-9 activities were quantified using the colorimetric caspase-1 assay kit (Abcam, Cambridge, MA, USA) and caspase-9 assay kit (Sigma-Aldrich, St. Louis, MO, USA), respectively. Briefly, cell lysates from IBV-infected and uninfected control chicken macrophages were incubated with specific chromogenic substrates (YVAD-pNA for caspase-1 and LEHD-pNA for caspase-9) [19]. The release of p-nitroaniline (pNA) was measured in 5 µg of cellular protein at 415 nm using a microplate reader (680 XR, Bio-Rad, Hercules, CA, USA), with enzymatic activity normalized to protein concentration. All steps followed the manufacturers’ protocols.

### 2.8. NF-κB Activation

Activation of NF-κB was measured using the IKK-α (Phospho-Ser176)/IKK-β (Phospho-Ser177) Cell-Based ELISA Kit (Biomatik Corporation, Cambridge, ON, Canada). Cells were cultured at a density of 20,000 cells per well in 96-well plates, fixed in situ, and phosphorylated IKK-α and IKK-β were detected using specific phospho-antibodies. A secondary HRP-conjugated antibody was applied, and the colorimetric signal was developed with substrate solution. Absorbance was recorded at 450 nm using an ELISA plate reader. Results were normalized to total protein content and expressed as relative NF-κB activation.

### 2.9. Detection of Apoptosis/Necroptosis by Immunofluorescence

To detect necroptosis in macrophages, cells were seeded on coverslips in 12-well plates at a density of 1 × 10^6^ cells/mL. IBV virions were adsorbed onto the macrophages for 1 h to allow initial viral attachment before removing unbound virus [20]. Following adsorption, cells were treated with a caspase-1/4 inhibitor to promote necroptosis and incubated for 24 h at 40 °C. Cells were washed with dPBS, fixed with 4% cold paraformaldehyde for 30 min, and stained with Alexa Fluor 488-conjugated antibody (Thermo Fisher Scientific, Waltham, MA, USA) for cytoskeletal visualization (1:400) and 10 µg/mL propidium iodide (Thermo Fisher Scientific, Waltham, MA, USA) for 30 min. After washing, coverslips were mounted with 4′,6-diamidino-2-phenylindole (DAPI) containing anti-fade media to preserve fluorescence. Cells were visualized using the Nikon Spectral Confocal Microscopy System (Nikon Corporation, Tokyo, Japan).

### 2.10. Statistical Analysis

Statistical analyses were performed using GraphPad Prism software (version 10.4.1, GraphPad Software, San Diego, CA, USA). A two-way ANOVA was applied for experiments involving two IBV strains or treatments across multiple time points, while one-way ANOVA was used for experiments with two strains or treatments at a single time point. Bonferroni’s post-hoc test was applied for multiple comparisons, and statistical significance was set at *p* < 0.05.

## 3. Results

### 3.1. Impact of Serum Concentration on Viral Shedding and Genome Load During IBV Infection in Chicken Macrophages

**Figure 1:**
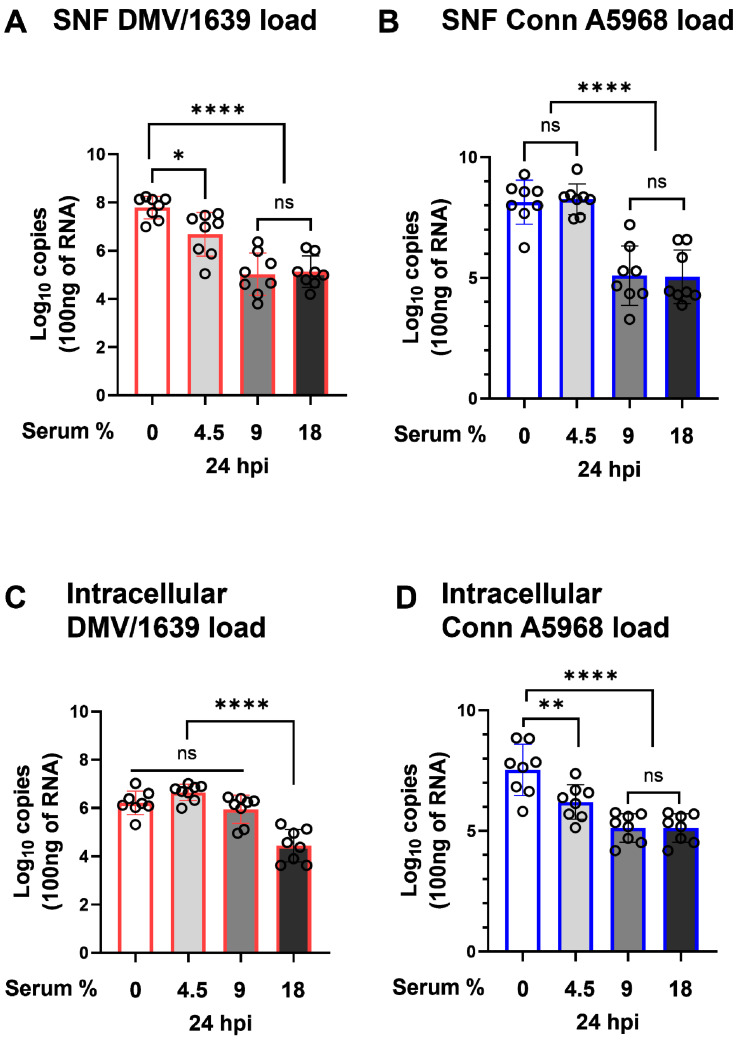
Effect of serum concentration on IBV genome load in chicken macrophages. Chicken macrophages were infected with IBV DMV/1639 or Conn A5968 strains for 1 h to allow viral adsorption. After infection, cells were cultured for 24 h in complete macrophage culture medium (18% serum) or media with reduced serum concentrations of 9% (50% reduction), 4.5% (75% reduction), or 0% serum. Viral genome loads in the culture supernatants (SNF) and cell lysates (**A**–**D**) for both strains were quantified by reverse transcription quantitative PCR (RT-qPCR). Statistical significance was determined using one-way ANOVA followed by Bonferroni’s post-hoc test. Data are presented as mean ± SD from two independent experiments (n = 4 replicates per experiment). ns = *p* > 0.05, while asterisks indicate statistically significant differences (* *p* < 0.05, ** *p* < 0.01, **** *p* < 0.0001) between between infected and mock conditions relative to the full culture medium (18% serum) or 100% serum mock-treated cells.

The viral genome load in SNF and cells was assessed 24 h hpi under four serum concentration conditions: complete media (18% serum), 50% media (9% serum), 25% media (4.5% serum), and serum-free media (Figure 1). In IBV DMV-infected cells, viral shedding increased with decreasing serum concentrations (Figure 1A). Compared to complete media, a modest increase in viral shedding was observed with 50% serum reduction (9% serum; *p* = 0.2), while a significant increase occurred with 25% serum reduction (4.5% serum; *p* < 0.001). However, no significant change was noted in serum-free conditions (*p* = 0.6). A similar trend was observed in IBV Conn A5968-infected cells, where reduced serum concentrations correlated with increased viral loads. A 50% serum reduction (9% serum) significantly elevated viral genome load (*p* = 0.03), and a 25% media reduction (4.5% serum) resulted in a highly significant increase (*p* < 0.0001), with no significant change to serum-free conditions (*p* > 0.9) (Figure 1B). These findings highlight the critical role of serum concentration in regulating viral shedding, with a more pronounced effect observed for the IBV Conn A5968 strain.

On the other hand, intracellular viral genome load exhibited a significant dependence on serum concentration in the culture media. For IBV DMV/1639, a substantial increase in intracellular viral genome load was observed beginning with 50% media (9% serum), showing a highly significant difference compared to complete media (18% serum) (Figure 1C; *p* < 0.0001). A further reduction in serum concentration to 25% media (4.5% serum) maintained this elevated intracellular viral genome load, but no additional significant changes were observed in serum-free conditions. For IBV Conn A5968, the intracellular viral genome load followed a similar pattern, with a marked increase first appearing at 25% media (4.5% serum) compared to complete media (Figure 1D; *p* < 0.0001). Unlike IBV DMV/1639, IBV Conn A5968 maintained high intracellular viral genome loads even under serum-free conditions, although no further significant differences were detected beyond 25% media.

These results emphasize the critical influence of serum concentration in the culture media on intracellular viral replication. Reduced serum levels create an environment that facilitates viral genome replication within infected cells, with IBV DMV/1639 and Conn A5968 strains responding to different serum thresholds. This highlights serum concentration as a pivotal factor in shaping the dynamics of intracellular viral genome load during IBV infection.

### 3.2. Impact of Serum Concentration on Cell Viability, Apoptosis, and Necroptosis in IBV-Infected Chicken Macrophages

**Figure 2:**
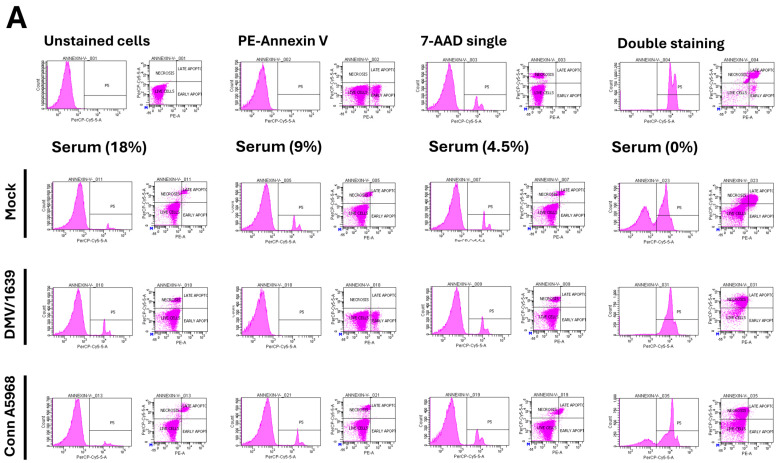

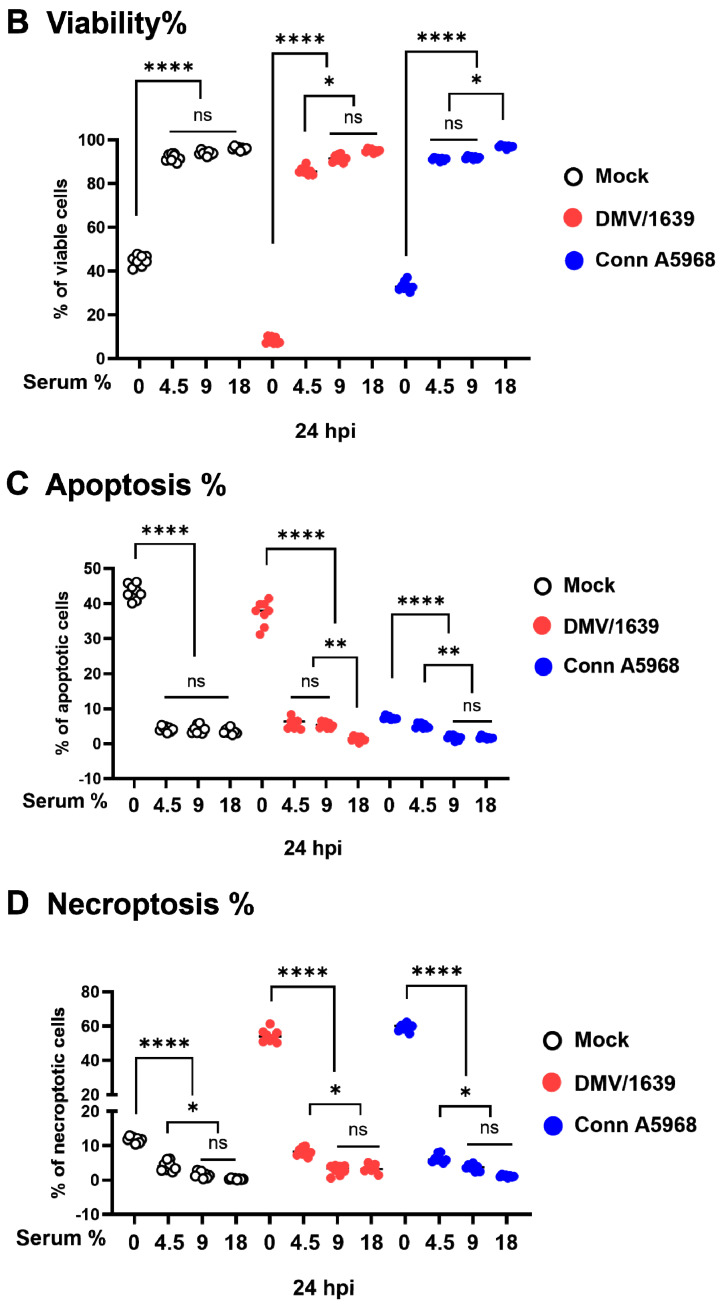
Effect of serum concentration on cell viability, apoptosis, and necroptosis in chicken macrophages. Chicken macrophages were exposed to IBV DMV/1639 or Conn A5968 strains for 1 h to allow viral adsorption. Subsequently, cells were cultured for 24 h in complete macrophage culture medium (18% serum) or media with reduced serum concentrations of 9% (50% reduction), 4.5% (75% reduction), or 0% serum. (**A**) The histograms and scatter plots in flow cytometry for the total cell population display unstained cells, single staining with PE-Annexin V or 7-AAD, and double staining (from left to right). The impact of serum concentration on cell viability, apoptosis, and necroptosis is shown in mock-infected, DMV/1639-infected, and Conn A5968-infected chicken macrophages. (**B**–**D**) Cell viability, apoptosis, and necroptosis were assessed by flow cytometry using the PE-Annexin V Apoptosis Detection Kit. Statistical analysis was performed using one-way ANOVA followed by Bonferroni post hoc tests. Data represent two independent experiments (n = 4 replicates per experiment) and are presented as mean ± SD. Asterisks indicate statistically significant differences (* *p* < 0.05, ** *p* < 0.01, **** *p* < 0.0001) between infected and mock-treated cells in the full culture medium (18% serum) or 100% serum conditions.

We next evaluated the effects of serum concentration on cell viability, apoptosis, and necroptosis at 24 hpi with IBV strains DMV/1639 and Conn A5968 (Figure 2). The gating strategy for determining the percentages of viable, apoptotic, and necroptotic cells using the PE-Annexin V apoptosis detection kit through flow cytometry analysis is detailed in Supplementary Figure S1.

The effect of all used caspase inhibitors on macrophage viability was evaluated using the MTT assay (Figure S2). Caspase-1/4, -3, and -9 inhibitors were tested at concentrations of 0, 1, 5, 10, 20, 50, and 100 µg/mL, while the pan-caspase inhibitor z-VAD-FMK was tested at 0, 1, 5, 10, 20, 50, and 100 µM. A notable reduction in cell viability was observed at concentrations ≥20 µg/mL for caspase-1/4, -3, and -9 inhibitors (Figure S2A,B,D) and ≥50 µM for z-VAD-FMK (Figure S1E). Based on these findings, the selected doses for subsequent experiments were 10 µg/mL for caspase inhibitors and 10 µM for z-VAD-FMK.

In mock-infected cells, serum-free conditions resulted in a significant decrease in cell viability compared to full serum, 50%, and 25% conditions (Figure 2B, *p* < 0.0001). However, no significant change in viability was observed with 50% (9% serum) or 25% (4.5% serum) conditions (*p* = 0.4 and *p* = 0.1, respectively). A marked reduction in cell viability was consistently observed following infection with both IBV strains under serum-free media, while no significant impact on viability was observed in the full serum media (*p* > 0.05) (Figure 2B).

Apoptosis, as measured by the combined percentages of early and late apoptotic cells, decreased in a dose-dependent manner as serum concentration was increased. In mock-infected cells, apoptosis significantly reduced from full serum to 9% serum (*p* < 0.02), 4.5% serum (*p* < 0.01), and was highly significant under serum-free conditions (*p* < 0.001) (Figure 2C). Infection with both IBV strains enhanced apoptosis in 25% (4.5% serum) and serum-free media compared to mock-infected cells (*p* < 0.0001 for both strains). However, no significant change in apoptosis was observed at 50% serum (*p* = 0.33 for IBV DMV/1639; *p* > 0.9 for IBV Conn A5968) or in full serum culture media (*p* > 0.09 for both strains) (Figure 2C). Apoptosis consistently decreased with increasing serum concentration in the media for both IBV strains, with diminished apoptosis observed in full serum culture media (18% serum) (Figure 2C). Apoptosis levels were minimal in full serum conditions (*p* > 0.09 for both strains). These findings suggest that higher serum concentrations in the media mitigate apoptosis during IBV infection.

Regarding necroptosis, in mock-infected cells, serum-free media significantly enhanced necroptotic cell death compared to full serum conditions (Figure 2D; *p* < 0.0001). However, no significant increase in necroptosis was observed with 9% or 4.5% serum conditions (*p* = 0.7 and *p* > 0.9, respectively). Both IBV strains induced a significant increase in necroptosis under serum-free conditions (*p* < 0.0001), and a lesser but significant increase was observed at 4.5% serum (*p* < 0.001 for IBV DMV/1639 and *p* = 0.03 for IBV Conn A5968). In contrast, no significant effect on necroptosis was observed at 9% serum or in full serum media (*p* > 0.9) (Figure 2D).

Overall, these findings demonstrate that reduced serum concentrations in culture media significantly increase viral genome load, decrease cell viability, and enhance both apoptosis and necroptosis in IBV DMV/1639 and IBV Conn A5968-infected macrophages, with the most pronounced effects observed under serum-free conditions.

### 3.3. Effects of Apoptosis Inhibition on IBV Genome Load in Macrophages

#### 3.3.1. Apoptosis Inhibition and Viral Genome Load

**Figure 3:**
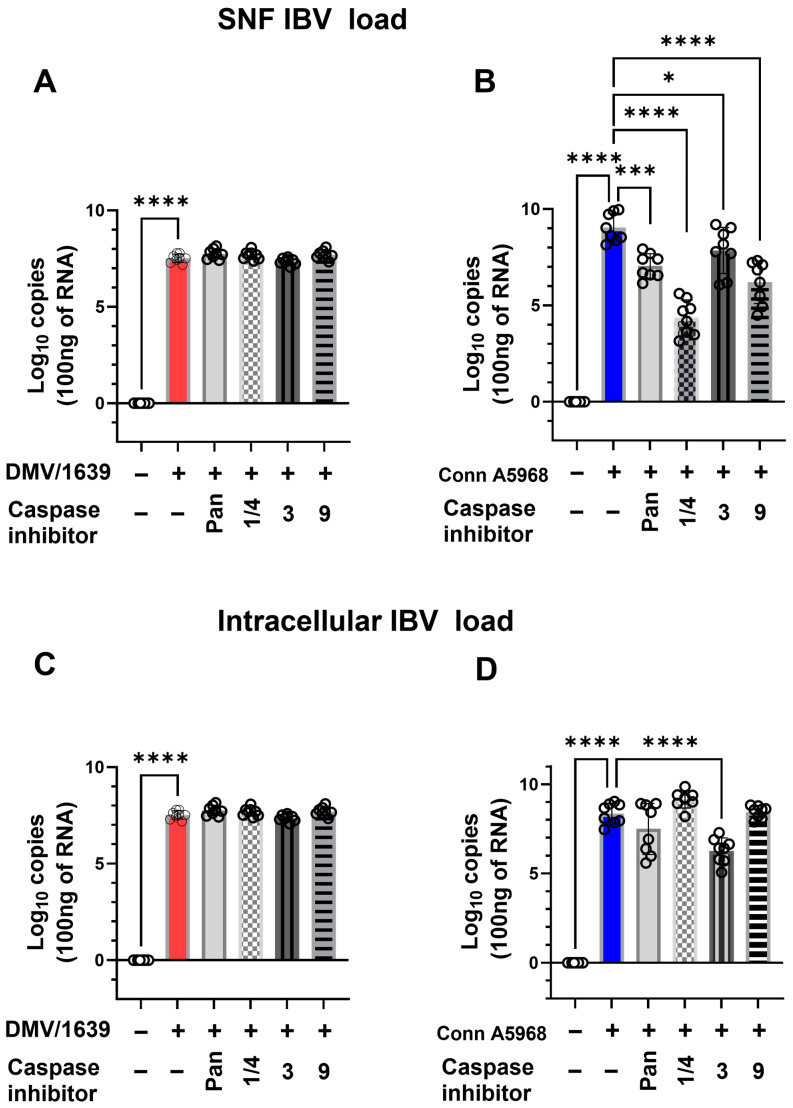
Effect of caspase inhibitors on IBV genome load in chicken macrophages. Chicken macrophages were exposed to IBV DMV/1639 or Conn A5968 strains for 1 h to facilitate viral adsorption. Afterward, IBV-infected chicken macrophages were co-treated for 24 h with specific caspase inhibitors (10 µg/mL for caspase-1/4, caspase-3, and caspase-9 inhibitors; 25 µM for the pan-caspase inhibitor Z-VAD-FMK) in macrophage culture medium containing 4.5% serum. Viral genome loads in the culture supernatant (SNF) and cell lysates were quantified by reverse transcription quantitative PCR (RT-qPCR). Statistical analysis was conducted using one-way ANOVA followed by Bonferroni post hoc tests. Data represent two independent experiments (n = 4 replicates per experiment) and are presented as mean ± SD. Means were compared to untreated infected cells, and asterisks indicate statistically significant differences (* *p* < 0.05, *** *p* < 0.001, **** *p* < 0.0001). (**A**): SNF IBV load in chicken macrophages after 1 h of exposure to DMV/1639; (**B**): SNF IBV load in chicken macrophages after 1 h of exposure to Conn A5968 strains; (**C**): Cell lysates IBV load in chicken macrophages after 1 h of exposure to DMV/1639; (**D**): Cell lysates IBV load in chicken macrophages after 1 h of exposure to Conn A5968 strains.

The impact of apoptosis inhibition on viral genome load was assessed both intracellularly and in SNF following treatment with specific caspase inhibitors, including caspase-1/4, -3, -9, and the pan-caspase inhibitor z-VAD-FMK (Figure 3). For IBV DMV/1639, no significant changes in intracellular viral genome load were observed after 24 h of treatment with any of the caspase inhibitors, including the pan-caspase inhibitor z-VAD-FMK (*p* > 0.05, Figure 3A,C). However, extracellular viral genome load in the SNF was significantly reduced with the pan-caspase inhibitor (*p* < 0.0001) and the caspase-9 inhibitor (*p* = 0.03, Figure 3A). In the case of IBV Conn A5968, treatment with all caspase inhibitors, including z-VAD-FMK, significantly decreased extracellular viral genome load (*p* < 0.0001 to *p* = 0.002, Figure 3D). The caspase-3 inhibitor showed only a minimal effect on extracellular viral genome load (*p* = 0.04).

For intracellular viral genome load in both IBV strains (Figure 3C,D), all used inhibitors had no significant effect, except for the caspase-3 inhibitor in IBV Conn A5968-infected cells, which caused a significant reduction (*p* < 0.001, Figure 3D). These findings indicate that apoptosis inhibition can modulate extracellular viral genome load more significantly than intracellular load, with the most pronounced effects observed using pan-caspase and caspase-9 inhibitors. This highlights a potential role for apoptosis-related pathways in viral shedding during IBV infection.

#### 3.3.2. Effects of Caspase Inhibition on Cell Viability, Apoptosis, and Necroptosis in IBV DMV/1639-Infected Cells

The effects of the pan-caspase inhibitor (z-VAD-FMK) on cell viability, apoptosis, and necroptosis were assessed in mock- and IBV-infected chicken macrophages under serum-free conditions (Supplementary Figure S3). After 24 h, treatment with 10 µM z-VAD-FMK significantly increased cell viability and reduced apoptosis in mock-infected cells (Figure S3A,B; *p* < 0.0001) but had no effect on necroptosis (Figure S3C). In IBV-infected chicken macrophages, z-VAD-FMK restored cell viability to levels exceeding those of mock-infected, untreated cells for both strains (Figure S3A). Its effects on apoptosis were strain-dependent; it reduced apoptosis in IBV DMV/1639-infected cells compared to untreated infected controls but increased apoptosis in IBV Conn A5968-infected cells (Figure S3B; *p* < 0.0001). Additionally, the inhibitor markedly reduced necroptosis in both IBV strains (Figure S3C; *p* < 0.0001). These findings highlight the dual and strain-specific effects of z-VAD-FMK on apoptotic pathways while consistently enhancing cell viability and mitigating necroptosis during IBV infection.

To mitigate the apoptotic effects of serum-free media on non-infected cells, we evaluated cell viability, apoptosis, and necroptosis in mock- and IBV-infected chicken macrophages cultured in 4.5% serum. This evaluation included the application of individual caspase inhibitors alongside the broad-spectrum pan-caspase inhibitor z-VAD-FMK. These conditions allowed us to better assess the modulatory effects of caspase inhibition on cell survival and death pathways in a more physiologically relevant serum environment. Furthermore, we investigated the effects of 24-h treatment with caspase inhibitors (1/4, 3, 8, 9, and pan-caspase) on cell viability, apoptosis, and necroptosis in mock-infected chicken macrophages cultured under 4.5% serum conditions (Figure S4). No significant changes were observed in the percentages of viable, apoptotic, or necroptotic cells following treatment with any of the inhibitors (Figure S4A–C).

**Figure 4:**
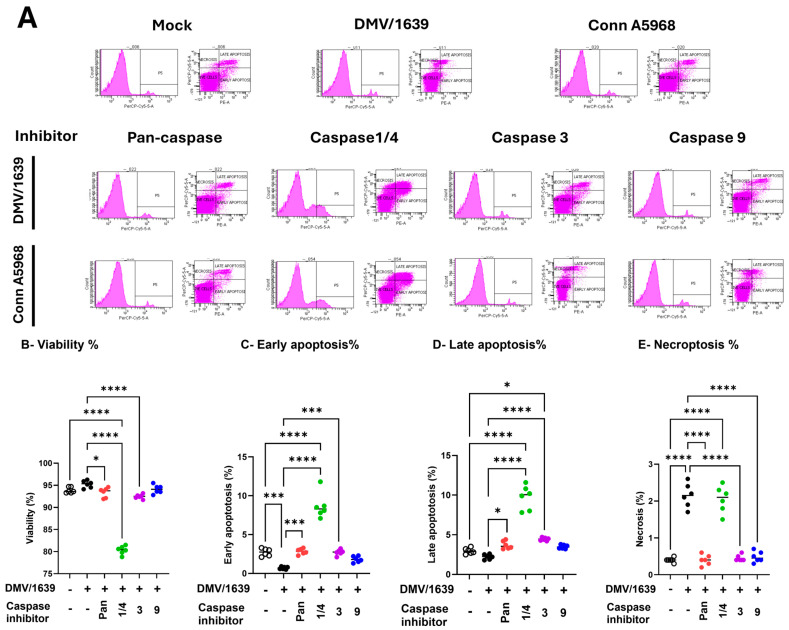

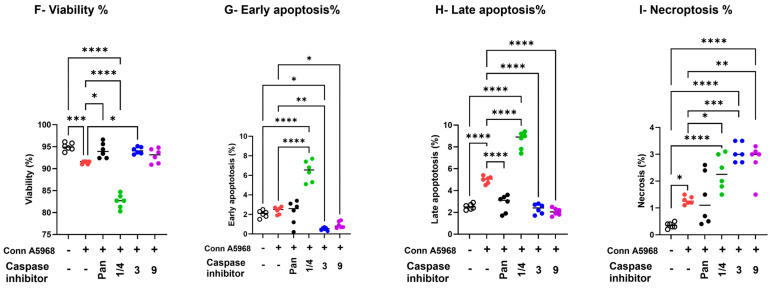
Effects of caspase inhibitors on cell viability, apoptosis, and necroptosis in chicken macrophages. Chicken macrophages were infected with IBV DMV/1639 (**B**–**E**) or Conn A5968 (**F**–**I**) strains at a multiplicity of infection (MOI) of 0.1 for 1 h to allow viral adsorption. Following infection, cells were treated with specific caspase inhibitors: 10 µg/mL for caspase-1/4, -3, and -9 inhibitors, and 25 µM for the pan-caspase inhibitor Z-VAD-FMK, for 24 h in culture medium containing 4.5% serum. (**A**) The histograms and scatter plots from flow cytometry for the total cell population illustrate the impact of caspase inhibitors on cell viability, early apoptosis, late apoptosis, and necroptosis are shown in DMV/1639-infected and Conn A5968-infected chicken macrophages. The percentages of viable cells (**B**,**F**), early apoptotic cells (**C**,**G**), late apoptotic cells (**D**,**H**), and necroptotic cells (**E**,**I**) were assessed using flow cytometry with the PE-Annexin V apoptosis detection kit. Data are presented as mean ± SD from two independent experiments (n = 3 replicates per experiment). Statistical significance was determined by one-way ANOVA followed by Bonferroni post hoc tests. Comparisons were made between treated, untreated infected cells, and mock infected cells, with asterisks indicating statistically significant differences (* *p* < 0.05, ** *p* < 0.01, *** *p* < 0.001, **** *p* < 0.0001).

Under 4.5% serum conditions, IBV DMV/1639 infection did not significantly affect cell viability compared to mock-infected controls (Figure 4B). However, early apoptosis was significantly decreased, while necroptosis was elevated in infected cells compared to controls (Figure 4C,E; *p* < 0.001). At 24 hpi, IBV DMV/1639 infection did not significantly impact early or late apoptosis (*p* > 0.05), but necroptosis was markedly elevated (Figure 4E; *p* < 0.0001). The pan-caspase inhibitor (z-VAD-FMK; 10 µM) had no significant effect on cell viability or apoptosis in IBV DMV/1639-infected macrophages (Figure 4B–D; *p* > 0.05) compared to control but increased both early and late apoptosis versus DMV/1639-infected cells (Figure 4C,D; *p* < 0.001, *p* < 0.05, respectively), while significantly reducing necroptosis (Figure 4E; *p* < 0.0001). In contrast, treatment with a caspase-1/4 inhibitor (10 µg/mL) significantly decreased cell viability in infected cells compared to untreated controls (Figure 4B; *p* < 0.0001). Caspase-1/4 inhibition significantly increased early and late apoptosis compared to DMV/1639-infected and non-infected controls (Figure 4C,D; *p* < 0.001) but had no effect on necroptosis in IBV DMV/1639-infected cells. Interestingly, caspase-3 inhibition did not significantly alter cell viability, enhanced early and late apoptosis, and reduced necroptosis compared to DMV/1639-infected cells (Figure 4C–E; *p* < 0.0001). The caspase-9 inhibitor had no significant impact on viability, early, or late apoptosis, but reduced necroptosis in IBV DMV/1639-infected cells (Figure 4E; *p* < 0.0001).

To sum up, IBV DMV/1639 infection primarily induces necroptosis without significantly affecting apoptosis; selective caspase inhibition differentially regulates cell death pathways, with caspase-1/4 inhibition exacerbating apoptosis, and caspase-3, -9, and pan-caspase inhibition mitigating necroptosis.

The impact of IBV Conn A5968 infection on chicken macrophages was marked by significantly reduced cell viability and increased late apoptosis and necroptosis (Figure 4F–I; *p* < 0.001, *p* < 0.0001, and *p* < 0.05, respectively). The pan-caspase inhibitor increased cell viability, reduced late apoptosis (Figure 4E,G; *p* < 0.05 and *p* < 0.0001, respectively), and had no effect on either early apoptosis or necroptosis compared to IBV Conn A5968-infected cells (Figure 4F,H). Caspase-1/4 inhibition further intensified these effects, leading to a significant reduction in viability and a marked increase in apoptosis and necroptosis (Figure 4F–I; *p* < 0.0001 each). Treatment with either caspase-3 or 9 inhibitors regained the cell viability in Conn A5968-infected cells, mitigated early and late apoptosis (Figure 4G,H), but paradoxically increased necroptosis in Conn A5968-infected macrophages (*p* < 0.001 for both).

Taken together, caspase inhibition affected IBV DMV/1639 and IBV Conn A5968 infections differently. In DMV/1639-infected chicken macrophages, necroptosis was dominant, with the pan-caspase and caspase-3/9 inhibitors reducing necroptosis, while caspase-1/4 inhibition increased apoptosis without affecting necroptosis. In Conn A5968-infected cells, both apoptosis and necroptosis were elevated, with the pan-caspase inhibitor improving viability and reducing late apoptosis, whereas caspase-1/4 inhibition further decreased viability and increased both apoptosis and necroptosis. Notably, caspase-3/9 inhibitors restored viability and reduced apoptosis but paradoxically increased necroptosis in Conn A5968-infected macrophages.

### 3.4. Cyclooxygenase-2/Prostaglandin E2 (COX-2/PGE2) Pathway Manipulation Alters Necroptosis and Cell Viability in IBV-Infected Chicken Macrophages

**Figure 5:**
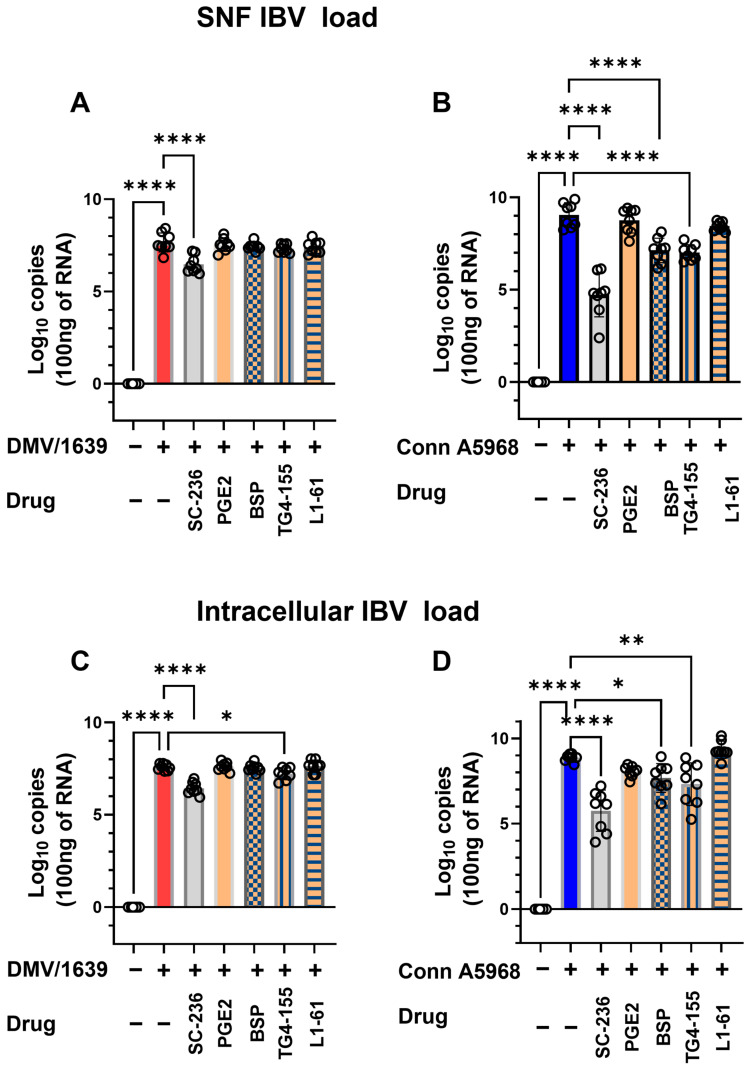
Impact of cyclooxygenase-2/ prostaglandin E2 (COX-2/PGE2) manipulation on IBV genome load in chicken macrophages. Chicken macrophages were infected with IBV DMV/1639 or Conn A5968 strains for 1 h to allow viral adsorption. Following infection, cells were co-treated with the cyclooxygenase-2 (COX-2) inhibitor SC-236 (10 µg/mL), exogenous PGE2 (10 µg/mL), the PGE2 inhibitor bromosulfophthalein (BSP, 25 µM), or antagonists targeting PGE2 receptors EP2 (TG4-155, 4 µM) and EP4 (L1-61, 8 µM) for 24 h in macrophage culture medium containing 4.5% serum. Viral genome loads were quantified in the culture supernatants (**A**,**B**) and cell lysates (**C**,**D**) using reverse transcription quantitative PCR (RT-qPCR). Statistical analysis was performed using one-way ANOVA followed by Bonferroni post hoc tests. Data represent two independent experiments (n = 4 replicates per experiment) and are expressed as mean ± SD. Comparisons were made to untreated infected cells, with asterisks indicating statistically significant differences (* *p* < 0.05, ** *p* < 0.01, **** *p* < 0.0001).

The impact of COX-2/PGE2 pathway manipulation on viral genome load was assessed both intracellularly and in SNF under conditions with 4.5% serum concentration (Figure 5). Treatment with COX-2 (SC-236) for 24 h significantly reduced the SNF viral genome load of the IBV DMV/1639 strain (*p* < 0.001, Figure 5A). Additionally, a significant decrease in intracellular viral genome load was observed following COX-2 inhibitor treatment (*p* < 0.001) and EP2 inhibition (*p* = 0.03, Figure 5C). For the IBV Conn A5968 strain, significant reductions in SNF viral genome load were observed with COX-2, EP2 (TG4-155), and PGE2 transporter (BSP) inhibitors (*p* < 0.0001 for all, Figure 5B). Similarly, intracellular viral genome load was significantly reduced by these inhibitors (*p* < 0.0001, *p* = 0.01, and *p* = 0.02, respectively, Figure 5D).

These findings demonstrate that manipulation of the COX-2/PGE2 pathway effectively reduces intracellular viral genome loads in both IBV strains. Additionally, selective reductions in extracellular viral genome load were observed in the DMV/1639 strain, underscoring the differential impact of these inhibitors on viral replication and underlying mechanisms.

**Figure 6:**
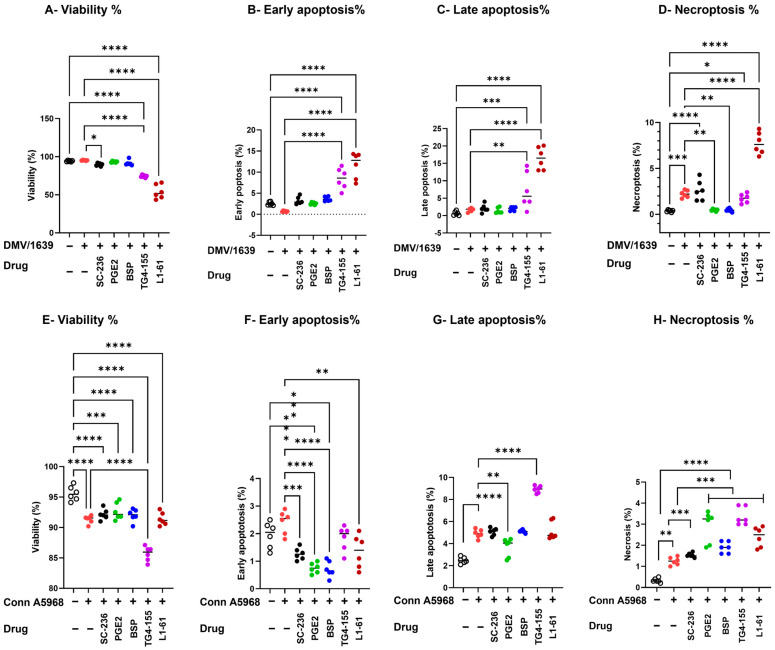
Impact of cyclooxygenase-2/prostaglandin E2 (COX-2/PGE2) manipulation on viability, apoptosis, and necroptosis in chicken macrophages. Chicken macrophages were infected with IBV DMV/1639 (**A**–**C**) or Conn A5968 (**D**–**F**) strains at a multiplicity of infection (MOI) of 0.1 for 1 h to allow viral adsorption. Following infection, cells were co-treated with the cyclooxygenase-2 (COX-2) inhibitor SC-236 (10 µg/mL), exogenous PGE2 (10 µg/mL), the PGE2 inhibitor bromosulfophthalein (BSP, 25 µM), or antagonists targeting PGE2 receptors EP2 (TG4-155, 4 µM) and EP4 (L1-61, 8 µM) for 24 h in culture medium containing 4.5% serum. The percentages of viable cells (**A**,**D**), early apoptotic cells (**B**,**F**), late apoptotic cells (**C**,**G**), and necroptotic cells (**D**,**H**), and necroptotic cells (**C**,**F**) were evaluated by flow cytometric analysis using the PE-Annexin V apoptosis detection kit. Statistical analysis was performed using one-way ANOVA followed by Bonferroni post hoc tests. Data represent two independent experiments (n = 3 replicates per experiment) and are expressed as mean ± SD. Comparisons were made between treated cells, untreated infected cells, and mock-infected controls, with asterisks denoting statistically significant differences (* *p* < 0.05, ** *p* < 0.01, *** *p* < 0.001, **** *p* < 0.0001).

The enhanced necroptosis induced by IBV DMV/1639 was reversed by co-treatment with COX-2 inhibitors and PGE2 pathway modulators (exogenous PGE2 and BSP) (*p* > 0.05), but unaffected by EP2 receptor and EP4 receptor inhibitors significantly increased necroptosis (TG4-155, L1-61; *p* < 0.05, *p* < 0.0001, Figure 6D, respectively).

Modulating the COX-2/PGE2 pathway significantly influenced cell viability, apoptosis, and necroptosis in IBV-infected chicken macrophages under 4.5% serum conditions (Figure 6). IBV DMV/1639 infection (Figure 6A–D) predominantly induced necroptosis (*p* < 0.001) without significantly affecting cell viability, early apoptosis, or late apoptosis (*p* > 0.05). COX-2 inhibition (SC-236) did not alter cell viability or apoptosis but significantly reduced necroptosis compared to untreated infected cells (*p* = 0.045). PGE2 receptor inhibitors (TG4-155 and L1-161) significantly decreased cell viability (*p* = 0.0005 and *p* < 0.0001, respectively). Conversely, exogenous PGE2 and PGE2-release inhibitors had no effect on cell viability or apoptosis (*p* > 0.05) but significantly reduced necroptosis in IBV DMV/1639-infected cells (*p* < 0.01 each, Figure 6A–D). Notably, the enhanced necroptosis associated with IBV DMV/1639 infection was reversed by co-treatment with COX-2 inhibitors and PGE2 pathway modulators (exogenous PGE2 and BSP) (*p* > 0.05), whereas EP2 and EP4 receptor inhibition (TG4-155, L1-161) significantly exacerbated necroptosis (*p* < 0.05 and *p* < 0.0001, respectively, Figure 6D).

In contrast, infection with IBV Conn A5968 (Figure 6E–H) significantly reduced cell viability (*p* < 0.001), while markedly increasing late apoptosis (*p* < 0.001) and necrosis (*p* < 0.01), without affecting early apoptosis (Figure 6F). Treatment with the EP2 inhibitor TG4-155 augmented these effects (*p* < 0.0001). Furthermore, COX-2 inhibitors, exogenous PGE2, BSP, and the EP4 inhibitor L1-161 enhanced necroptosis in Conn A5968-infected chicken macrophages (*p* = 0.01, *p* < 0.0001, *p* = 0.0001, *p* = 0.0001, respectively). These findings suggest that manipulation of the COX-2/PGE2 pathway has varied effects on IBV strain-specific outcomes in cell viability, apoptosis, and necroptosis.

Taken together, these findings highlight the critical role of the COX-2/PGE2 pathway in modulating IBV strain-specific effects of IBV on macrophage survival, with DMV/1639 infection primarily inducing necroptosis, which was mitigated by COX-2 inhibition and PGE2 pathway modulators, whereas Conn A5968 infection led to significant cell death via apoptosis and necrosis, further exacerbated by COX-2 and PGE2 receptor inhibitors.

### 3.5. Impact of Inhibition of NLRP3 Inflammasome and RIPK1 on Cell Viability, Apoptosis, and Necroptosis in IBV-Infected Chicken Macrophages

**Figure 7:**
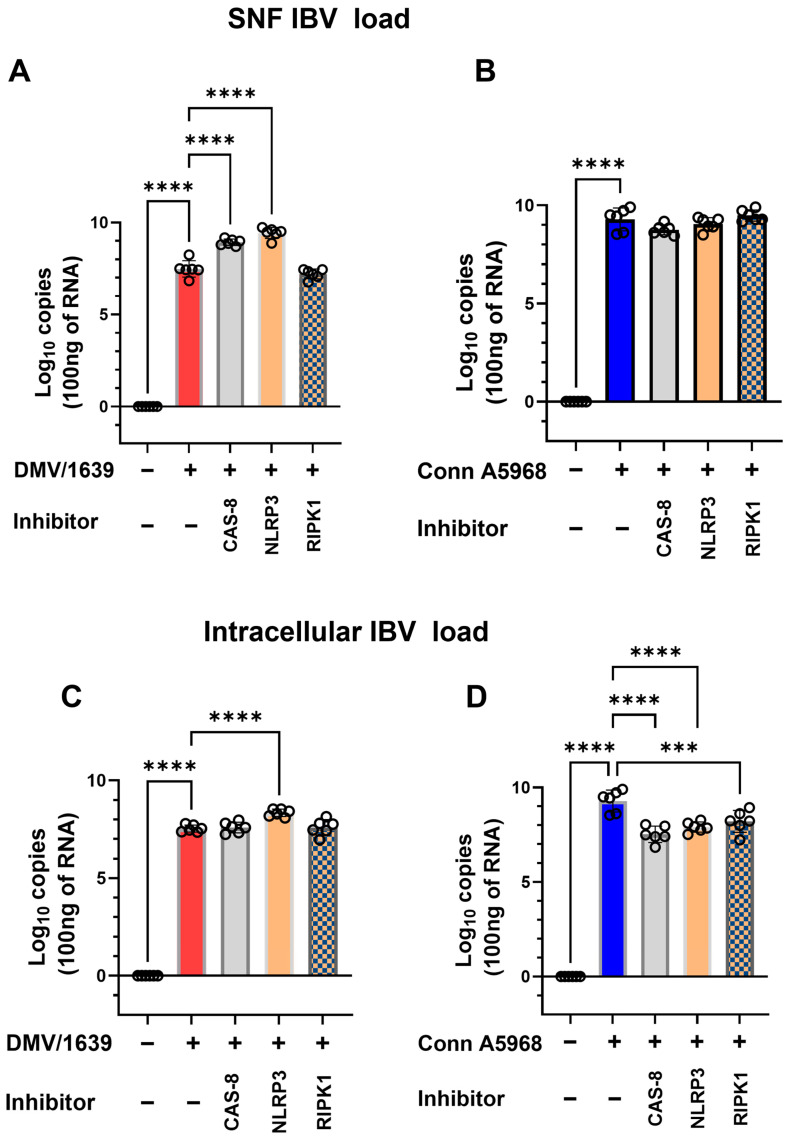
Impact of caspase-8, NLRP3, and RIPK1 inhibitors on IBV genome load in chicken macrophages. Chicken macrophages were infected with IBV DMV/1639 (**A**,**C**) or Conn A5968 (**B**,**D**) strains at a multiplicity of infection (MOI) of 0.1 for 1 h to allow viral adsorption. Following infection, cells were co-treated with a caspase-8 inhibitor (Z-IETD-FMK; 20 µM), an NLRP3 inflammasome inhibitor (MCC950; 10 µM), or a RIPK1 inhibitor (Necrostatin-1; 25 µM) for 24 h in culture medium containing 4.5% serum. Viral genome loads were quantified in both cell lysates and culture supernatant fluid (SNF). Statistical analysis was performed using one-way ANOVA followed by Bonferroni post hoc tests. Data represent two independent experiments (n = 3 replicates per experiment) and are expressed as mean ± SD. Comparisons were made to untreated infected cells, with asterisks indicating statistically significant differences (*** *p* < 0.001, **** *p* < 0.0001).

This experiment was conducted to evaluate the effects of caspase-8, NLRP3 inflammasome, and RIPK1 inhibitors on viral genome load, cell viability, apoptosis, and necroptosis in chicken macrophages infected with two strains of IBV (DMV/1639 and Conn A5968) to assess strain-specific responses to these treatments. Firstly, IBV genome load was assessed intracellularly and in culture SNF (Figure 7). In macrophages infected with IBV DMV/1639, treatment with a caspase-8 inhibitor (Z-IETD-FMK; 20 µM) or an NLRP3 inflammasome inhibitor (MCC950; 10 µM) significantly increased viral genome load in the SNF after 24 h (Figure 7A; *p* < 0.0001). However, no significant changes were observed with a RIPK1 inhibitor (Necrostatin-1; 25 µM). In contrast, these inhibitors did not alter the viral genome load in the SNF of IBV Conn A5968-infected macrophages (Figure 7B). Regarding intracellular viral genome load, treatment with the NLRP3 inflammasome inhibitor significantly enhanced viral load in IBV DMV/1639-infected cells (Figure 7C; *p* < 0.0001), while caspase-8 and RIPK1 inhibitors had no significant effect. Conversely, in IBV Conn A5968-infected cells, all three inhibitors (caspase-8, NLRP3 inflammasome, and RIPK1) significantly reduced intracellular viral load (Figure 7D; *p* < 0.0001 for each). These findings demonstrate that the effects of caspase-8, NLRP3 inflammasome, and RIPK1 inhibitors on viral genome load in IBV-infected chicken macrophages were strain-specific. In DMV/1639-infected cells, NLRP3 inflammasome and caspase-8 inhibitors significantly increased viral genome load in the supernatant, while RIPK1 inhibition had no effect. In Conn A5968-infected cells, all three inhibitors significantly reduced intracellular viral genome load without altering the supernatant load. These results highlight distinct host–pathogen interactions for DMV/1639 and Conn A5968 strains.

**Figure 8:**
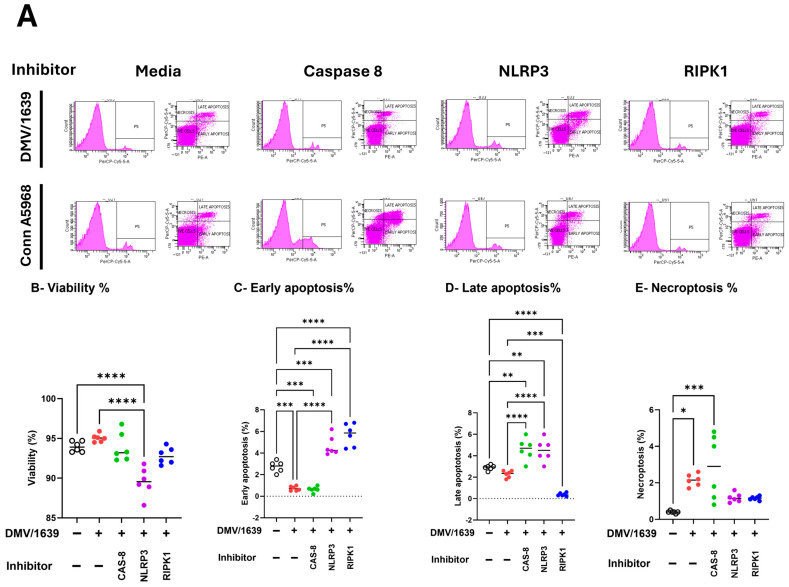

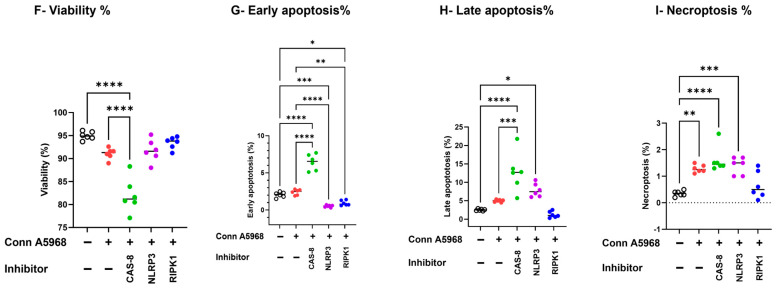
Impact of caspase-8, NLRP3, and RIPK1 inhibitors on cell viability, apoptosis, and necroptosis in chicken macrophages. Chicken macrophages were infected with IBV DMV/1639 (**B**–**E**) or Conn A5968 (**F**–**I**) strain at a multiplicity of infection (MOI) of 0.1 for 1 h to allow viral adsorption. Following infection, cells were treated with a caspase-8 inhibitor (Z-IETD-FMK; 20 µM), an NLRP3 inflammasome inhibitor (MCC950; 10 µM), or a RIPK1 inhibitor (Necrostatin-1; 25 µM) for 24 h in culture medium containing 4.5% serum. (**A**) The histograms and scatter plots from flow cytometry illustrate the impact of these inhibitors on cell viability, apoptosis, and necroptosis in DMV/1639-infected and Conn A5968-infected chicken macrophages. The percentages of viable cells (**B**,**F**), early apoptotic cells (**C**,**G**), late apoptotic (**D**,**H**), and necroptotic cells (**E**,**I**) were evaluated to assess the effects of these treatments on cell viability, early and late apoptosis, and necroptosis were evaluated. Statistical analysis was performed using one-way ANOVA followed by Bonferroni post hoc tests. Data represent two independent experiments (n = 3 replicates per experiment) and are expressed as mean ± SD. Comparisons were made between treated cells, untreated infected cells, and mock-infected controls, with asterisks denoting statistically significant differences (* *p* < 0.05, ** *p* < 0.01, *** *p* < 0.001, **** *p* < 0.0001).

Then the effects of caspase-8, NLRP3 inflammasome, and RIPK1 inhibitors on cell viability, apoptosis, and necroptosis (Figure 8). In IBV DMV/1639-infected macrophages, treatment with a caspase-8 inhibitor had no significant impact on cell viability or total apoptosis in Supplementary Figure S5; hence, it reduced early apoptosis and increased late apoptosis, *p* < 0.001 versus control cells, respectively, while significantly increasing necroptosis (Figure 8E; *p* < 0.01). Conversely, the NLRP3 inflammasome inhibitor significantly reduced cell viability and enhanced apoptosis (Figure 8B,C; *p* < 0.001 and *p* < 0.0001, respectively), but did not affect necroptosis compared to infected cells and control non-treated cells (Figure 8D). RIPK1 inhibition led to decreased cell viability, increased early apoptosis, and reduced late apoptosis (Figure 8B,C; *p* < 0.0001, *p* < 0.05, respectively), thus increasing total apoptosis (Figure S5) without altering necroptosis compared to infected and control non-treated cells (Figure 8E). In IBV Conn A5968-infected macrophages, the caspase-8 inhibitor significantly reduced cell viability and increased total apoptosis (both early and late) as well as necroptosis (Figure 8F–I; *p* < 0.0001, *p* < 0.0001, *p* < 0.0001, *p* < 0.05, respectively). The NLRP3 inflammasome inhibitor did not affect cell viability and apoptosis compared to infected and mock-infected cells, while significantly enhancing necroptosis compared to control non-infected cells (Figure 8E–G; *p* < 0.05). However, RIPK1 inhibition did not affect cell viability, significantly reduced early apoptosis without affecting late apoptosis (Figure 8G,H; *p* < 0.01), and showed no effect on necroptosis.

These findings demonstrate that caspase-8, NLRP3 inflammasome, and RIPK1 inhibitors significantly influenced viral genome load, apoptosis, necroptosis, and cell viability; the effects varied between the DMV/1639 and Conn A5968 strains. In summary, the results demonstrate that in DMV/1639-infected cells, caspase-8 inhibition reduced necroptosis, NLRP3 inflammasome inhibition decreased cell viability and increased apoptosis, and RIPK1 inhibition reduced viability and increased apoptosis without affecting necroptosis. In Conn A5968-infected cells, caspase-8 inhibition increased both apoptosis and necroptosis, NLRP3 inflammasome inhibition enhanced necroptosis, and RIPK1 inhibition reduced apoptosis without impacting viability or necroptosis.

**Figure 9:**
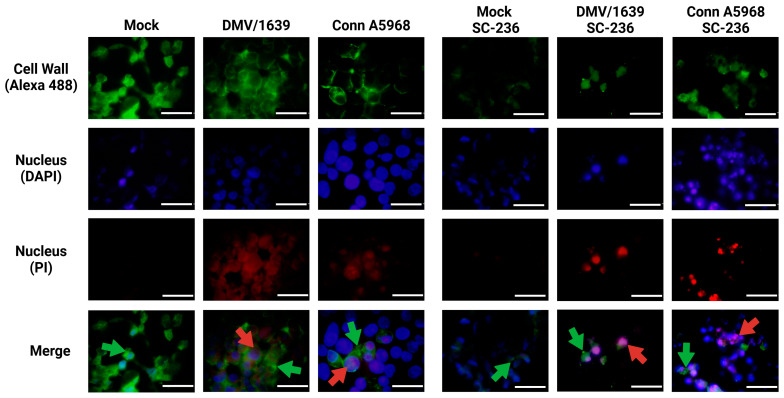

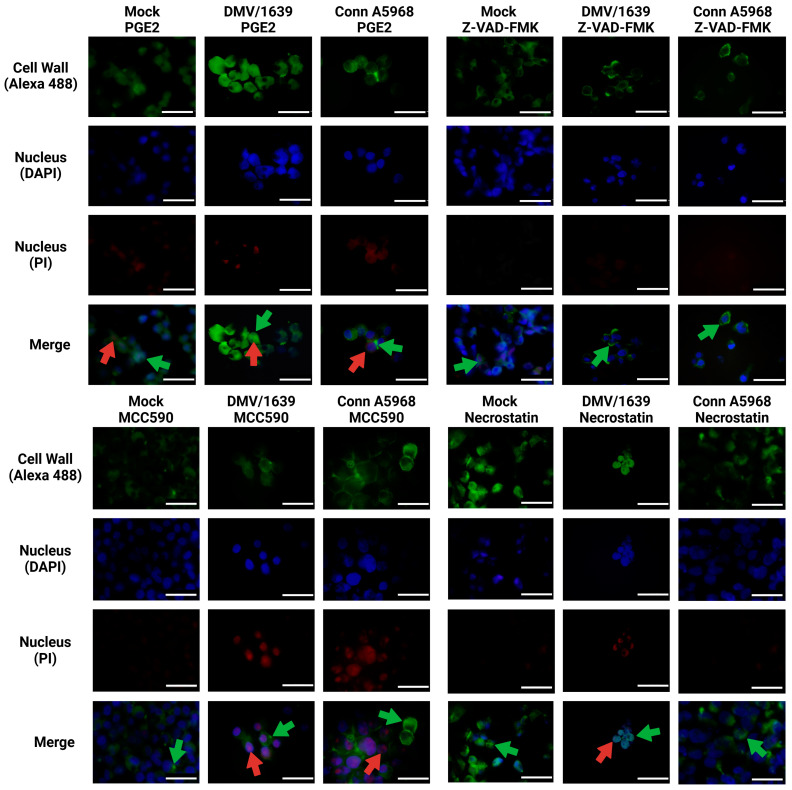
Immunofluorescence analysis of chicken macrophages treated with inhibitors targeting COX-2, pan-caspase, NLRP3, and RIPK1 following IBV infection. Chicken macrophages were infected with IBV DMV/1639 or Conn A5968 strains at a multiplicity of infection (MOI) of 0.1 for 1 h to facilitate viral adsorption. Following infection, cells were treated with a COX-2 inhibitor (SC-236; 10 µg/mL), exogenous prostaglandin E2 (PGE2; 10 µg/mL), a pan-caspase inhibitor (Z-VAD-FMK; 25 µM), an NLRP3 inflammasome inhibitor (MCC950; 10 µM), or a RIPK1 inhibitor (Necrostatin-1; 25 µM) for 24 h in culture medium containing 4.5% serum. Immunofluorescence staining was performed to visualize the cell wall (Alexa Fluor 488; green arrows), nuclei (DAPI; blue), and necrotic nuclei (propidium iodide; red arrows). Representative images from two independent experiments are shown. Scale bar = 50 µm, magnification 100×.

The results in Figure 9 demonstrate the effects of inhibitors targeting COX-2, pan-caspase, NLRP3 inflammasome, and RIPK1 on necroptosis in IBV-infected chicken macrophages using immunofluorescence analysis. Representative images were obtained from chicken macrophages infected with IBV DMV/1639 or Conn A5968 strains (MOI = 0.1) and treated with the indicated inhibitors for 24 h post-infection. Immunofluorescence staining revealed cell wall structures (green, Alexa Fluor 488), nuclei (blue, DAPI), and necrotic nuclei (red, PI). Scale bar = 50 µm; magnification = 100×. Treatment with the COX-2 inhibitor SC-236 significantly enhanced necroptosis, as evidenced by an increased number of propidium iodide (PI)-stained necrotic nuclei (red, indicated by white arrows). In contrast, exogenous prostaglandin E2 (PGE2) treatment did not affect necroptosis, showing negligible PI staining compared to the untreated control. Interestingly, inhibition of the NLRP3 inflammasome with MCC950 also enhanced necroptosis, comparable to the effects observed with SC-236. However, treatment with the RIPK1 inhibitor Necrostatin-1 effectively inhibited necroptosis, as evidenced by the marked reduction in PI-stained necrotic nuclei. These findings suggest that RIPK1 plays a critical role in mediating necroptosis during IBV infection, whereas COX-2 and NLRP3 pathways modulate necroptosis differently.

This analysis provides mechanistic insights into the modulation of necroptosis in IBV-infected chicken macrophages and highlights potential therapeutic targets for mitigating excessive necroptotic cell death.

### 3.6. Impact of COX-2/PGE2, Caspases, NLRP3 Inflammasome, and RIPK1 Pathways on Caspase-1, Caspase-3, and NF-κB Activities

The activities of caspase-1, caspase-9, and NF-κB were measured to evaluate their roles in the inflammatory programmed cell death (PCD) of chicken macrophages during IBV infection., and how these key mediators are regulated under various conditions, including treatment with pharmacological inhibitors, to better characterize their involvement in the cellular response to IBV infection.

#### 3.6.1. Regulation of Caspase-1 Activity by NLRP3 Inflammasome and RIPK1 Pathways in Chicken Macrophages During IBV Infection

**Figure 10:**
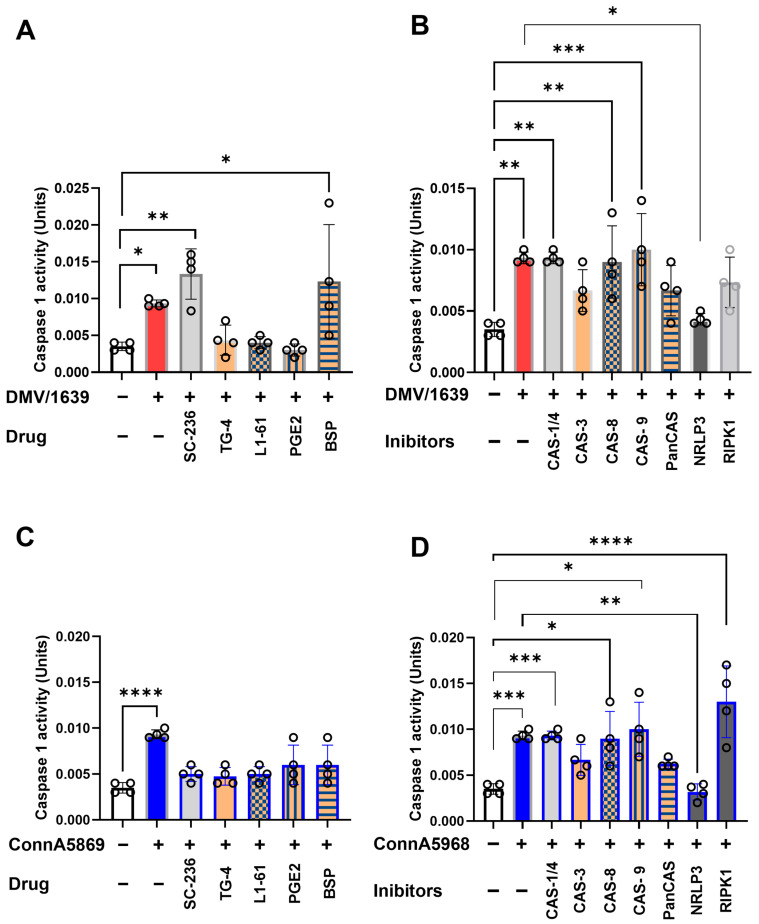
Impact of various inhibitors on caspase-1 activity in chicken macrophages. Chicken macrophages were infected with IBV DMV/1639 (**A**,**B**) or Conn A5968 (**C**,**D**) strains at a multiplicity of infection (MOI) of 0.1 for 1 h to allow viral adsorption. Following infection, cells were treated with inhibitors targeting caspase-1/4, caspase-3 (10 µM), caspase-8, caspase-9, broad-spectrum caspases (Z-VAD-FMK; 25 µM), the NLRP3 inflammasome (MCC950; 10 µM), RIPK1 (Necrostatin-1; 25 µM), COX-2 (SC-236; 10 µg/mL), exogenous PGE2 (10 µg/mL), the PGE2 transporter (BSP; 25 µM), and EP receptor antagonists (EP2: TG4-155; 4 µM and EP4: L1-61; 8 µM) for 24 h in culture medium containing 4.5% serum. Caspase-1 activity, normalized to 5 µg of protein, was measured colorimetrically at 415 nm using the caspase-1 colorimetric assay. Statistical analyses were conducted using one-way ANOVA followed by Bonferroni post hoc tests. Data represent four biological replicates and are expressed as mean ± SD. Comparisons were made to untreated infected cells, with asterisks indicating statistically significant differences (* *p* < 0.05, ** *p* < 0.01, *** *p* < 0.001, **** *p* < 0.0001).

This experiment aimed to assess the effects of various pharmacological inhibitors on intracellular caspase-1 activity, a key mediator in the inflammatory and apoptotic pathways following IBV infection. Chicken macrophages were treated with LPS (10 µg/mL) for either 6 or 24 h. LPS treatment significantly enhanced caspase-1 activity compared to PBS-treated controls (Supplementary Figure S7A). Additionally, the impact of these inhibitors on caspase-9 activity was evaluated in mock-infected cells. Among the tested inhibitors, caspase-3, -8, and -9 inhibitors, as well as the EP4 receptor antagonist (L1-61), the NLRP3 inflammasome inhibitor, and exogenous PGE2, all significantly enhanced caspase-9 activity (Supplementary Figure S7B, *p* < 0.05). In IBV DMV/1639-infected macrophages, intracellular caspase-1 activity was significantly increased compared to mock-infected controls at 24 hpi (Figure 10A). This elevated activity was further augmented by treatment with the COX-2 inhibitor SC-236 (*p* < 0.01) and BSP (*p* < 0.05), whereas no significant effect was observed with exogenous PGE2, its receptor antagonist TG4-155, or L-161 BSP. When DMV/1639-infected macrophages were treated with inhibitors targeting caspases, NLRP3 inflammasome, and RIPK1, the caspase-1 activity was notably increased by caspase-1, caspase-8, and caspase-9 inhibitors (*p* < 0.01) (Figure 10B). In contrast, pan-caspase and RIPK1 inhibitors had no significant effect. However, treatment with the NLRP3 inflammasome inhibitor significantly reduced caspase-1 activity (*p* < 0.05) (Figure 10B). Similarly, IBV Conn A5968 infection significantly enhanced caspase-1 activity in chicken macrophages at 24 hpi (*p* < 0.0001) (Figure 10C). This increase was unaffected by inhibitors targeting COX-2, exogenous PGE2, its receptor antagonist TG4-155, or L-161 BSP. Additionally, inhibitors of caspases-1, -3, -8, and -9, as well as pan-caspase inhibitors, did not alter the elevated activity. Notably, treatment with the NLRP3 inflammasome inhibitor significantly reduced caspase-1 activity (*p* < 0.01) (Figure 10D), while the RIPK1 inhibitor markedly enhanced caspase-1 activity (*p* < 0.05) (Figure 10D).

#### 3.6.2. Regulation of Caspase-9 Activity by NLRP3 Inflammasome and RIPK1 Pathways in Chicken Macrophages During IBV Infection

Caspase-9 activity, a key marker of the intrinsic apoptotic pathway triggered by internal cellular stress (e.g., DNA damage, oxidative stress), was evaluated in IBV-infected chicken macrophages. To induce DNA damage, chicken macrophages were exposed to UV light (365 nm, 10 mJ/cm²) for 10 min, and caspase-9 activity was measured at 6 and 24 h post-exposure (Supplementary Figure S7C). Additionally, the effects of various drugs and inhibitors on caspase-9 activity were assessed in mock-infected cells. Among the tested inhibitors, only caspase-3, -8, and -9 inhibitors, along with the EP2 receptor antagonist (L1-61), significantly enhanced caspase-9 activity (Supplementary Figure S7D, *p* < 0.05).

**Figure 11:**
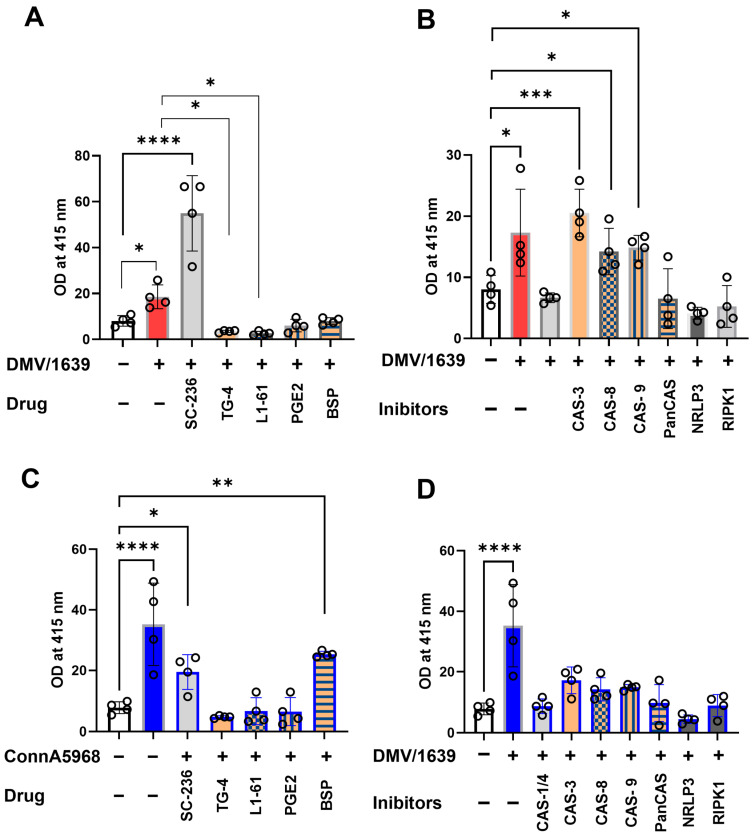
Impact of various inhibitors on caspase-9 activity in chicken macrophages. Chicken macrophages were infected with IBV DMV/1639 (**A**,**B**) or Conn A5968 (**C**,**D**) strains at a multiplicity of infection (MOI) of 0.1 for 1 h to facilitate viral adsorption. Following infection, cells were treated with inhibitors targeting caspase-9, caspase-1/4, caspase-3 (10 µM), caspase-8, broad-spectrum caspases (Z-VAD-FMK; 25 µM), the NLRP3 inflammasome (MCC950; 10 µM), RIPK1 (Necrostatin-1; 25 µM), COX-2 (SC-236; 10 µg/mL), exogenous PGE2 (10 µg/mL), the PGE2 transporter (BSP; 25 µM), and EP receptor antagonists (EP2: TG4-155; 4 µM and EP4: L1-61; 8 µM) for 24 h in culture medium containing 4.5% serum. Caspase-9 activity, normalized to 5 µg of protein, was measured colorimetrically at 415 nm using the caspase-9 colorimetric assay. Statistical analyses were conducted using one-way ANOVA followed by Bonferroni post hoc tests. Data represent four biological replicates and are presented as mean ± SD. Comparisons were made to untreated infected cells, with asterisks indicating statistically significant differences (* *p* < 0.05, ** *p* < 0.01, *** *p* < 0.001, **** *p* < 0.0001).

In IBV DMV/1639-infected macrophages, intracellular caspase-9 activity was significantly increased compared to mock-infected controls at 24 hpi (Figure 11A). This elevated activity was further augmented by treatment with the COX-2 inhibitor SC-236 (*p* < 0.01) and BSP (*p* < 0.05), whereas no significant effect was observed with exogenous PGE2, its receptor antagonist TG4-155, or L-161 BSP. When DMV/1639-infected macrophages were treated with inhibitors targeting caspases, NLRP3 inflammasome, and RIPK1, the caspase-1 activity was notably increased by caspase-1, caspase-8, and caspase-9 inhibitors (*p* < 0.01) (Figure 11B). In contrast, pan-caspase and RIPK1 inhibitors had no significant effect. However, treatment with the NLRP3 inflammasome inhibitor significantly reduced caspase-1 activity (*p* < 0.05) (Figure 11B). Similarly, IBV Conn A5968 infection significantly enhanced caspase-1 activity in chicken macrophages at 24 hpi (*p* < 0.0001) (Figure 11C). This increase was unaffected by inhibitors targeting COX-2, exogenous PGE2, its receptor antagonist TG4-155, or L-161 BSP. Additionally, inhibitors of caspases-1, -3, -8, and -9, as well as pan-caspase inhibitors, did not alter the elevated activity. Notably, treatment with the NLRP3 inflammasome inhibitor significantly reduced caspase-1 activity (*p* < 0.01) (Figure 11D), while the RIPK1 inhibitor markedly enhanced caspase-1 activity (*p* < 0.05) (Figure 11D).

Overall, these results highlight the critical role of NLRP3 inflammasome and RIPK1 signaling in IBV-induced immune responses, reinforcing their potential as therapeutic targets.

#### 3.6.3. Regulation of NF-κB Activation by COX-2/PGE2, NLRP3 Inflammasome, and RIPK1 Pathways in Chicken Macrophages During IBV Infection

The effects of various pharmacological inhibitors on NF-κB activation were analyzed to elucidate their regulatory roles in untreated and IBV-infected chicken macrophages. In untreated control macrophages, all tested inhibitors demonstrated some degree of reduction in NF-κB activation. However, significant inhibitory effects were observed with inhibitors targeting caspase-9, pan-caspase, the NLRP3 inflammasome (MCC950), RIPK1 (Necrostatin-1), COX-2 (SC-236), EP2 (TG4-155), EP4 (L1-61), the PGE2 transporter (BSP), and exogenous PGE2 (Supplementary Figure S7E, *p* < 0.0001).

**Figure 12:**
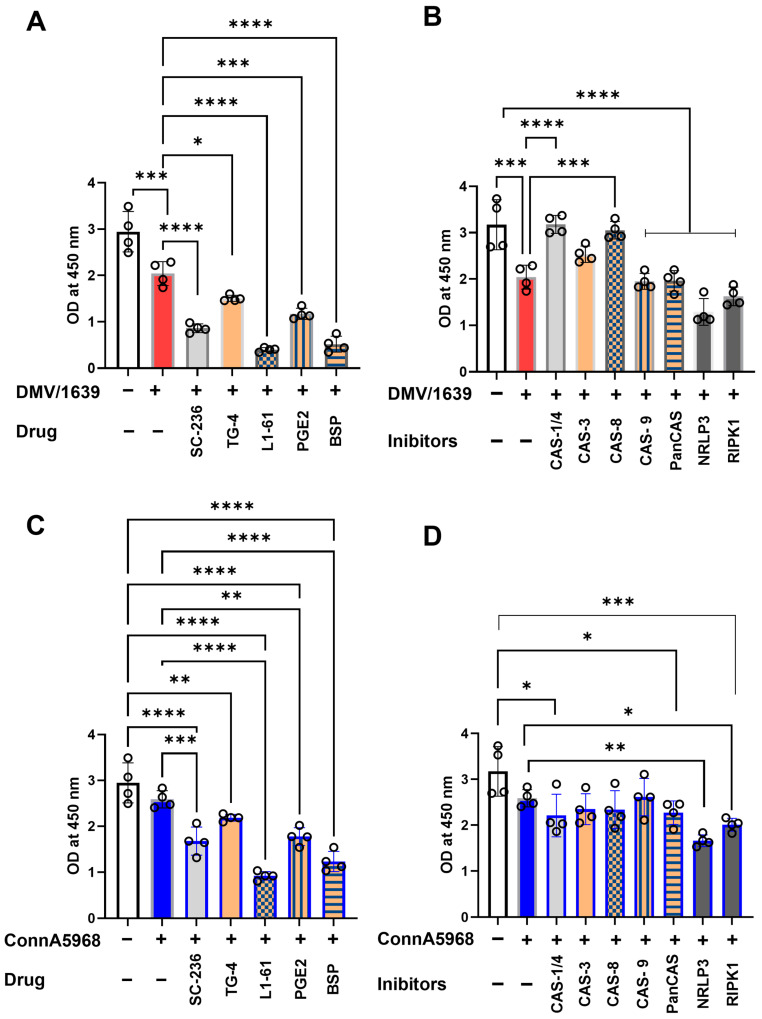
Impact of caspase, NLRP3, RIPK1, COX-2, PGE2, EP receptor, and PGE transporter inhibitors on NF-κB activation in chicken macrophages. Chicken macrophages were infected with IBV DMV/1639 (**A**,**C**) or Conn A5968 (**B**,**D**) strains at a multiplicity of infection (MOI) of 0.1 for 1 h to facilitate viral adsorption. Following infection, cells were treated with inhibitors targeting caspases-1/4, -3 (10 µM), -8, -9, and broad-spectrum caspases (Z-VAD-FMK; 25 µM), the NLRP3 inflammasome (MCC950; 10 µM), RIPK1 (Necrostatin-1; 25 µM) (**A**,**C**), or COX-2 inhibitor (SC-236; 10 µg/mL), exogenous PGE2 (10 µg/mL), the PGE2 transporter inhibitor (BSP; 25 µM), and EP receptor antagonists (EP2: TG4-155; 4 µM and EP4: L1-61; 8 µM) for 24 h in serum-free culture medium. NF-κB activation was quantified colorimetrically using the IKK-α (Phospho-Ser176)/IKK-β (Phospho-Ser177) Cell-Based ELISA assay, with measurements taken using an ELISA plate reader. Statistical analyses were performed using one-way ANOVA, followed by Bonferroni post hoc tests. Data represent four biological replicates and are presented as mean ± SD. Comparisons were made to untreated infected cells, with statistically significant differences indicated by asterisks (* *p* < 0.05, ** *p* < 0.01, *** *p* < 0.001, **** *p* < 0.0001).

In macrophages infected with IBV DMV/1639, NF-κB activation was significantly reduced at 24 hpi compared to uninfected controls (Figure 12A,B, *p* < 0.01). Pharmacological modulation of the COX-2/PGE2 pathway further inhibited NF-κB activation in infected cells, with varying levels of significance for the inhibitors: SC-236 (*p* < 0.001), TG4-155 (*p* < 0.05), L1-61 (*p* < 0.001), BSP (*p* < 0.001), and exogenous PGE2 (*p* < 0.001) (Figure 12A). Similarly, the effects of caspase inhibitors, NLRP3 inflammasome antagonists, and RIPK1 inhibitors were assessed. Antagonists of caspase-1 and caspase-8 significantly enhanced NF-κB activation (*p* < 0.001). In contrast, inhibitors targeting caspase-9, pan-caspases, the NLRP3 inflammasome, and RIPK1 significantly suppressed NF-κB activation in IBV DMV/1639-infected macrophages at 24 hpi (*p* < 0.001 for each) (Figure 12B).

In macrophages infected with the IBV Conn A5968 strain, no significant differences in NF-κB activation were observed between infected and non-infected cells. Nevertheless, inhibitors targeting COX-2, EP2, exogenous PGE2, and BSP significantly reduced NF-κB activation (Figure 12C; *p* < 0.001, *p* < 0.0001, *p* < 0.05, and *p* < 0.0001, respectively). While caspase inhibitors did not cause significant reductions, antagonists of the NLRP3 inflammasome and RIPK1 markedly suppressed NF-κB activation (Figure 12D; *p* < 0.01 and *p* < 0.05, respectively).

These findings identify the COX-2/PGE2 pathway, caspases, NLRP3 inflammasome, and RIPK1 as key regulators of NF-κB activation, suggesting specific drug targets to mitigate inflammation during IBV infection in chicken macrophages.

## 4. Discussion

Our findings provide compelling evidence that the interplay between viral replication, cell death pathways, and inflammatory mediators is intricately modulated by serum concentration, caspase activity, and the COX-2/PGE2 pathway in chicken macrophages infected with IBV. The observed increase in viral replication and shedding at reduced serum concentrations underscores the critical role of the extracellular environment in determining the efficiency of IBV replication, with both DMV/1639 and Conn A5968 strains displaying heightened viral loads under these conditions, albeit with strain-specific differences. Furthermore, the protective effects of broad-spectrum caspase inhibitors in reducing apoptosis and necroptosis highlight the pivotal role of caspase-mediated cell death pathways in IBV pathogenesis. Our data also reveal that manipulation of the COX-2/PGE2 pathway exerts divergent effects on cell viability and death, as well as on viral replication, depending on the IBV strain, thereby emphasizing its dual role in either mitigating or exacerbating disease outcomes. Collectively, these findings advance our understanding of IBV-induced macrophage responses and identify key molecular targets, such as caspases, the NLRP3 inflammasome, RIPK1, and the COX-2/PGE2 pathway, for intervention strategies in IBV infections.

The observed dependence of IBV replication and shedding on serum concentration provides novel insights into the role of extracellular factors in regulating viral dynamics in chicken macrophages. Reduced serum concentrations significantly enhanced viral shedding and intracellular genome loads, with notable differences between IBV DMV/1639 and Conn A5968 strains (Figure 1). These findings are consistent with previous studies reporting that serum components, including nutrients, growth factors, and regulatory proteins, influence viral replication by modulating cellular metabolism and immune responses. For instance, Firth et al. [20] demonstrated that serum deprivation alters metabolic pathways, leading to increased viral replication in other avian viruses. Interestingly, the distinct response thresholds for the two IBV strains suggest strain-specific interactions with host cells under serum-depleted conditions. IBV DMV/1639 showed significant changes in viral replication at higher serum concentrations (9%), while Conn A5968 required further serum reduction (4.5%) to achieve comparable intracellular genome loads. This observation aligns with the hypothesis that strain-specific differences in virulence and host tropism modulate sensitivity to extracellular cues, as suggested by Jackwood et al. [21]. Furthermore, the lack of additional increases in viral replication under serum-free conditions highlights a potential threshold effect, beyond which cellular machinery cannot further support viral genome replication. This plateau may result from the depletion of essential factors for viral replication or heightened cellular stress responses under extreme serum deprivation, as previously reported by Gonnella et al. [22]. These findings highlight the critical role of serum-derived factors in shaping IBV infection outcomes and support the hypothesis that modulation of extracellular environments can serve as a potential strategy to influence viral replication. Understanding the interplay between serum concentration and viral replication may provide valuable insights for optimizing in vitro infection models.

Our findings in Figure 2 highlight the important role of serum concentration in regulating cell viability, apoptosis, and necroptosis during IBV infection in macrophages. Reduced serum levels, particularly under serum-free conditions, significantly increased viral genome load, decreased cell viability, and enhanced both apoptosis and necroptosis. These results align with previous studies highlighting serum components’ role in modulating cell survival and death pathways during viral infections [23,24]. The observed dose-dependent reduction in apoptosis with higher serum concentrations suggests that serum proteins may inhibit apoptotic signaling, in line with earlier reports on serum’s protective effects against cell death [25]. Additionally, increased necroptosis in low serum conditions, especially in IBV DMV/1639-infected cells, supports the notion that serum depletion may promote necroptotic death, a mechanism implicated in viral pathogenesis [26]. The variation in responses between the two IBV strains points to strain-specific differences in immune modulation and their influence on macrophage cell death pathways [27,28]. These findings demonstrate that serum concentration critically influences macrophage responses during IBV infection, affecting viral replication and immune response, suggesting potential therapeutic strategies targeting serum components or cell death pathways to control IBV infection.

The results of apoptosis inhibition on viral genome load suggest a more pronounced effect on extracellular viral shedding compared to intracellular replication (Figure 3). Specifically, while caspase inhibition did not significantly affect intracellular viral genome load, both pan-caspase and caspase-9 inhibitors reduced the extracellular viral genome load in IBV-infected chicken macrophages. This is in line with previous studies indicating that apoptosis can influence viral shedding and extracellular virus release. Apoptotic pathways are known to play a critical role in modulating viral replication and release in various viral infections, and our data extend these findings to IBV, showing that inhibition of apoptosis can reduce viral shedding without affecting intracellular replication [29]. Interestingly, the impact of caspase inhibitors varied between the two IBV strains, with IBV DMV/1639 showing no significant change in intracellular viral genome load, while IBV Conn A5968 showed a significant reduction following caspase-3 inhibition. This strain-specific response could be attributed to differences in the virus-host interactions or the mechanisms of cell death and viral replication. Previous research has suggested that different strains of IBV may induce distinct patterns of apoptosis and necroptosis, which could influence how inhibitors affect viral replication and shedding [30]. The lack of effect on intracellular genome load, despite significant changes in extracellular viral load, may reflect the complex relationship between cell death and viral release. Moreover, the findings from caspase inhibition experiments on cell viability, apoptosis, and necroptosis support the hypothesis that caspase-mediated cell death may be integral to the regulation of macrophage responses during IBV infection. The pan-caspase inhibitor z-VAD-FMK consistently enhanced cell viability and reduced apoptosis in mock- and IBV-infected chicken macrophages under serum-free conditions. These results align with previous studies showing that pan-caspase inhibition can prevent cell death and promote cell survival, further confirming the pivotal role of apoptosis in the regulation of macrophage responses to viral infection [31]. However, the strain-dependent effects on apoptosis suggest the two IBV strains may modulate apoptotic pathways differently, thus influencing viral replication and pathogenesis. These findings emphasize the role of apoptosis in viral shedding and suggest that targeting apoptosis-related pathways may help reduce viral replication and tissue damage during IBV infection. Therefore, investigating the interplay between apoptosis, necroptosis, and viral replication across IBV strains is essential for understanding virus-host dynamics and developing drug targets.

The findings of the present study in Figure 4 reinforce the critical role of caspase activity in modulating cell death pathways during IBV infection in chicken macrophages. Previous studies have shown that caspase inhibitors can either promote or suppress specific forms of cell death, depending on the context and type of infection. Our results align with those of Liu et al. [32], who demonstrated that caspase-1/4 inhibition in influenza virus-infected cells exacerbates cell death, including increased apoptosis, similar to what we observed with IBV DMV/1639-infected macrophages. On the other hand, our study also revealed that caspase-3 and caspase-9 inhibitors, while alleviating apoptosis, paradoxically promoted necroptosis in IBV Conn A5968-infected cells, which is consistent with previous research showing that caspase inhibition can shift the balance toward necroptotic cell death in various viral infections [33]. Notably, pan-caspase inhibition, which blocks the activity of multiple caspases, reduced both apoptosis and necroptosis, supporting the idea that broad inhibition of caspase activity can provide protection against excessive cell death during viral infections, as suggested by studies on other RNA viruses such as Zika [34]. Furthermore, the differential effects observed between the two IBV strains (DMV/1639 and Conn A5968) in response to caspase inhibition highlight the strain-specific nature of apoptosis and necroptosis regulation. This supports the hypothesis that IBV strains may differentially modulate host cell death pathways to facilitate their replication and persistence in macrophages, as suggested by earlier work on viral strain variability and host–pathogen interactions [35]. The strain-specific modulation of cell death pathways could contribute to variations in viral replication and pathogenesis, as evidenced by the observed differences in cell viability, apoptosis, and necroptosis between the two IBV strains. Our findings suggest that caspase inhibition may help reduce cell death and tissue damage during IBV infection. However, strain-dependent effects require further studies of an extensive nature to understand the mechanisms behind IBV’s manipulation of CDP, guiding targeted strategies to control viral replication and limit host damage.

The manipulation of the COX-2/PGE2 pathway has been shown to significantly influence IBV infection outcomes, with differential effects observed between IBV strains. The findings in Figure 5, in line with our previous studies, indicate that inhibiting COX-2 and PGE2 receptors can reduce both intracellular and extracellular viral genome loads in IBV-infected chicken macrophages [11] and in tracheal organ culture [18]. Specifically, COX-2 and EP2 inhibitors effectively decreased viral replication in IBV DMV/1639 and IBV Conn A5968 strains, suggesting that modulation of this pathway may interfere with viral replication mechanisms. This aligns with previous reports, which demonstrated that COX-2 inhibition can impair viral replication in other infections, including influenza and human immunodeficiency virus (HIV) [5,36]. Moreover, the significant reduction of viral genome load in the supernatant of infected cells after COX-2 inhibition suggests that the COX-2/PGE2 pathway plays a pivotal role in viral shedding, which has not been widely explored in the context of IBV infection. The effects of COX-2/PGE2 manipulation on cell viability, apoptosis, and necroptosis further highlight the complexity of host immune responses to IBV infection (Figure 6). In the case of IBV DMV/1639, COX-2 inhibitors and PGE2 modulation effectively reduced necroptosis, a form of programmed necrosis that has been implicated in tissue damage and viral clearance during infections [37]. Interestingly, while PGE2 itself did not affect apoptosis or cell viability, it reduced necroptosis, which may represent a protective mechanism against excessive cell death and tissue damage during infection. Conversely, IBV Conn A5968 infection exhibited more pronounced effects on cell viability and apoptosis, with COX-2 and EP4 receptor inhibitors exacerbating necroptosis. These findings support the hypothesis that the COX-2/PGE2 axis may influence strain-specific host–pathogen interactions and immune responses [38]. The strain-specific effects observed in IBV infection highlight the complex role of the COX-2/PGE2 pathway in viral replication and cell death. Our findings suggest that while modulating this pathway can reduce viral replication and influence cell death, its effects vary across strains, reflecting an intricate host–pathogen interaction. These results align with previous studies showing context-dependent modulation of cell death and viral replication by the COX-2/PGE2 pathway in other respiratory viruses [39]. This suggests that targeting COX-2/PGE2 may be a promising therapeutic approach for IBV, though further research is needed to fully understand its molecular mechanisms and improve antiviral strategies.

The manipulation of the NLRP3 inflammasome, caspase-8, and RIPK1 pathways in IBV-infected chicken macrophages demonstrates distinct strain-specific effects on viral genome load, cell viability, apoptosis, and necroptosis (Figure 8). Notably, our results suggest that the impact of these inhibitors is highly dependent on the IBV strain, highlighting the complex interactions between viral factors and host immune responses. In IBV DMV/1639-infected macrophages, inhibition of the NLRP3 inflammasome significantly increased viral genome load, while caspase-8 inhibition reduced necroptosis without affecting apoptosis or cell viability. These findings contrast with previous studies in which NLRP3 inflammasome inhibition was reported to reduce viral replication in other viral infections, such as in studies of the influenza A virus, where NLRP3 inflammasome activation was shown to facilitate viral clearance [40]. Our observation that caspase-8 inhibition significantly reduced necroptosis in DMV/1639-infected macrophages aligns with the established role of caspase-8 as a key regulator of necroptotic pathways. This finding is consistent with studies in other viral infections, where caspase-8 inhibition has been shown to prevent the execution of necroptosis and reduce inflammation [41]. Interestingly, caspase-8 inhibition did not significantly impact cell viability or apoptosis in DMV/1639-infected cells, highlighting its selective modulation of necroptosis in this context. In Conn A5968-infected macrophages, caspase-8 inhibition exhibited distinct effects, leading to decreased cell viability and increased apoptosis and necroptosis. These findings are in line with evidence suggesting that caspase-8 serves as a central switch between apoptotic and necroptotic cell death mechanisms, where its inhibition shifts the balance toward enhanced necroptosis and apoptosis [42]. The NLRP3 inflammasome inhibitor demonstrated strain-dependent effects. In DMV/1639-infected macrophages, NLRP3 inhibition significantly reduced cell viability and enhanced apoptosis without affecting necroptosis. This indicates that NLRP3 activation may play a protective role in promoting cell survival and suppressing apoptosis during DMV/1639 infection. Conversely, in Conn A5968-infected cells, NLRP3 inhibition selectively enhanced necroptosis, suggesting a differential role for NLRP3 activation in regulating cell death pathways depending on the viral strain. RIPK1 inhibition in DMV/1639-infected macrophages significantly reduced cell viability and increased apoptosis but did not alter necroptosis, supporting RIPK1’s critical role in apoptotic regulation during infection. In Conn A5968-infected macrophages, RIPK1 inhibition reduced apoptosis without significantly affecting cell viability or necroptosis. These findings align with prior studies indicating that RIPK1 serves as a critical mediator of apoptotic and necroptotic pathways, with its functional impact varying depending on the cellular context and infection model [43]. Overall, our results demonstrate that pharmacological inhibition of caspase-8, NLRP3 inflammasome, and RIPK1 exerts distinct, strain-specific effects on cell viability, apoptosis, and necroptosis in IBV-infected chicken macrophages.

The differential effects observed with these inhibitors in the two IBV strains underscore the strain-specific nature of host–pathogen interactions and provide valuable insights into the mechanisms underlying cell death and viral replication. In particular, our findings suggest that while caspase-8 and NLRP3 inflammasome pathways may promote necroptosis in certain IBV strains, RIPK1 inhibition serves as a more potent strategy for reducing necroptosis. These results align with previous studies indicating that RIPK1 is a key driver of necroptosis and inflammation in various viral infections, including studies on the regulation of necroptosis during influenza and hepatitis B virus infections [44,45]. Furthermore, the effects of COX-2 inhibition and exogenous PGE2 treatment on necroptosis observed in the immunofluorescence analysis further support the involvement of these pathways in modulating cell death in IBV-infected chicken macrophages. Our findings show that modulation of caspase-8, NLRP3 inflammasome, and RIPK1 pathways affects IBV replication, cell death, and viral genome load, with strain-specific variations in necroptosis and apoptosis.

In macrophages infected with IBV Conn A5968, a similar increase in caspase-1 activity was observed, but this was not influenced by COX-2 inhibitors, exogenous PGE2, or PGE2 receptor antagonists, further indicating strain-specific differences in how these pathways modulate caspase-1 activity. Remarkably, the NLRP3 inflammasome inhibitor MCC950 significantly reduced caspase-1 activity, aligning with previous studies that have demonstrated a central role for NLRP3 in inflammasome activation during viral infections [46,47]. Furthermore, RIPK1 inhibition in IBV Conn A5968-infected cells enhanced caspase-1 activity, suggesting a potential crosstalk between RIPK1 and NLRP3 pathways in regulating caspase-1 activity, as described in other viral infection models [48]. These findings underscore the strain-dependent regulation of caspase-1 by inflammasome and RIPK1 signaling pathways and suggest that targeting these pathways could offer therapeutic potential for modulating inflammatory responses during IBV infection. In particular, the reduction in caspase-1 activity with NLRP3 inhibitors and the enhancement of caspase-1 with RIPK1 inhibition point to complex interactions between these pathways that may influence IBV-induced inflammation and tissue damage. These results are consistent with previous studies identifying NLRP3 and RIPK1 as critical regulators of inflammatory responses in viral infections [49,50]. The differential effects of these inhibitors across IBV strains also suggest that therapeutic strategies targeting these pathways may need to be tailored to specific viral strains to achieve the desired anti-inflammatory effects. The regulation of caspase-9 activity was also assessed, given its role in the intrinsic apoptotic pathway. As shown in Supplementary Figure S7, UV-induced DNA damage in chicken macrophages significantly enhanced caspase-9 activity, with inhibitors of caspases-3, -8, and -9, as well as the EP2 receptor antagonist L1-61, further elevating this activity. In IBV-infected chicken macrophages, caspase-9 activity was also significantly increased, particularly in IBV DMV/1639-infected cells (Figure 11), and was further augmented by COX-2 inhibition. These results suggest that the intrinsic apoptotic pathway may be activated in response to IBV infection, with potential modulation by COX-2/PGE2 signaling, as previously reported in other viral models [51]. Conversely, the lack of effect of RIPK1 inhibitors on caspase-9 activity indicates that RIPK1’s role in apoptosis may be distinct from its involvement in caspase-1 activation [50,51]. These data highlight the complex interactions between inflammasomes, caspases, and RIPK1 in regulating cell death during IBV infection, emphasizing the need for further exploration of these mechanisms to develop targeted therapies aimed at reducing IBV-induced inflammation and tissue damage.

The regulation of NF-κB activation by COX-2/PGE2, NLRP3 inflammasome, and RIPK1 signaling pathways is critical for understanding the inflammatory response during IBV infection. As shown in Figure 12, the pharmacological inhibition of these pathways demonstrated significant effects on NF-κB activation, underscoring their regulatory roles in inflammation. In untreated macrophages, the inhibition of caspase-9, pan-caspases, the NLRP3 inflammasome, RIPK1, COX-2, and PGE2 pathways notably reduced NF-κB activation (Supplementary Figure S7E), consistent with findings in other studies showing that these pathways modulate NF-κB signaling in various inflammatory contexts [52]. Specifically, the reduction of NF-κB activation by COX-2 inhibitors (e.g., SC-236) and PGE2 antagonists (e.g., BSP) is in line with previous work indicating the crucial role of COX-2 and PGE2 in controlling inflammation and immune responses during viral infections [52,53]. In IBV-infected chicken macrophages, we observed a significant decrease in NF-κB activation at 24 hpi, which was further diminished by modulation of the COX-2/PGE2 pathway using inhibitors, such as SC-236, TG4-155, and BSP (Figure 12). This is consistent with previous reports that COX-2/PGE2 signaling promotes NF-κB activation, which in turn facilitates the inflammatory response to viral infections [52,54]. In the case of the DMV/1639 strain, caspase-1 and caspase-8 inhibition increased NF-κB activation, suggesting a pro-inflammatory role of these caspases, which aligns with findings that caspase-8 activation can modulate NF-κB signaling and contribute to inflammation [55]. Conversely, inhibitors targeting caspase-9, pan-caspases, NLRP3 inflammasome, and RIPK1 significantly suppressed NF-κB activation, suggesting that these pathways are involved in the negative regulation of NF-κB activation during IBV infection, as previously suggested for other viral infections [56,57,58,59]. Interestingly, in macrophages infected with the IBV Conn A5968 strain, no significant changes in NF-κB activation were observed compared to uninfected controls, suggesting that strain-specific variations in NF-κB regulation may exist. This strain-dependent discrepancy in NF-κB activation could be attributed to differences in viral pathogenesis and the host’s immune response [60]. Nevertheless, the inhibition of COX-2, EP2, PGE2, and BSP in these cells still resulted in a reduction of NF-κB activation [61], highlighting the importance of these pathways in regulating inflammation even in the absence of significant changes in NF-κB levels.

Targeting the COX-2/PGE2 pathway presents a promising strategy for mitigating excessive inflammation in IBV-infected poultry. Excessive PGE2 production has been implicated in dysregulated immune responses and virus-induced tissue pathology [62,63]. Modulating this pathway could help balance the immune response by reducing IBV-induced tissue damage while preserving essential antiviral defenses [60]. Studies have shown that COX-2 inhibition can limit inflammatory damage and enhance immune homeostasis in respiratory viral infections [12,64]. In chickens, COX-2 inhibition with meloxicam reduced PGE2 production, alleviating immune suppression against Marek’s disease virus (MDV) infection and improving disease outcomes [65]. Additionally, controlling PGE2 signaling could mitigate excessive apoptosis and necroptosis, thereby reducing severe immunopathology and organ dysfunction [64]. Therefore, by fine-tuning COX-2/PGE2 activity, it may be possible to reduce IBV-associated mortality, limit viral replication, and minimize economic losses in poultry farms. Moreover, maintaining immune homeostasis through COX-2/PGE2 regulation could help lower the risk of secondary infections and associated complications, ultimately improving overall poultry health and productivity. However, modulating apoptosis, necroptosis, and inflammasome pathways for therapeutic intervention in poultry could present several potential risks. Modulating these pathways might inadvertently suppress essential immune responses, leading to increased susceptibility to secondary infections [66,67]. For instance, inhibiting apoptosis or necroptosis could prolong viral persistence by allowing infected cells to survive longer, thereby facilitating viral spread [68]. Conversely, excessive activation of cell death pathways could exacerbate tissue damage, impair organ function, and compromise overall poultry health [69,70]. Additionally, interventions affecting inflammasome activation could disrupt the delicate balance between protective immunity and immunopathology, potentially leading to either excessive inflammation or inadequate viral clearance [71,72]. These risks highlight the need for precise therapeutic modulation to achieve effective viral control without compromising immune homeostasis.

In a broader context, the present study emphasizes the complex interplay between viral replication, host cell death, and immune response regulation during IBV infection. By revealing strain-specific differences in viral replication, apoptosis, necroptosis, and inflammatory signaling, these findings offer new perspectives on viral pathogenesis and potential therapeutic targets. Modulation of serum concentration, cell death pathways, and key immune pathways, such as COX-2/PGE2 and NLRP3 inflammasome may provide promising strategies for controlling viral replication and alleviating tissue damage during IBV infections. Future research should aim to investigate the molecular mechanisms underlying these strain-specific responses, as well as the development of targeted therapies that address these pathways to reduce viral replication and enhance immune clearance. Moreover, studies exploring the role of extracellular factors in shaping viral dynamics across other avian viruses may broaden our understanding of virus-host interactions and inform broader antiviral strategies.

## 5. Conclusions

This study highlights the impact of serum concentration on viral replication and host immune responses in IBV-infected chicken macrophages, with strain-specific differences observed between IBV DMV/1639 and Conn A5968. Our results emphasize the roles of apoptosis, necroptosis, and inflammasome pathways in regulating viral replication and cell survival. Key signaling pathways, such as COX-2/PGE2, NLRP3 inflammasome, and RIPK1, present potential therapeutic targets for controlling viral replication and reducing tissue damage. Given the strain-dependent variations, further investigation into these molecular mechanisms is crucial for advancing antiviral therapies and improving in vitro models for studying IBV and other viral infections.

## Data Availability

The datasets used and/or analyzed within the frame of the study can be provided by the corresponding author upon reasonable request.

## Supplementary Materials

The following supporting information can be downloaded at: https://www.mdpi.com/article/10.3390/v17040503/s1.
