# Supplementary material for: Cyclooxygenase-2/Prostaglandin E2 Pathway Facilitates Infectious Bronchitis Virus-Induced Necroptosis in Chicken Macrophages, a Caspase-Independent Cell Death
Source: Viruses. 2025 Mar 31;17(4):503. doi: 10.3390/v17040503 (PMC12030959; doi:10.3390/v17040503)
Supplement: Supplementary file 1 [file viruses-17-00503-s001.zip › viruses-3480430-supplementary.pdf]

Supplementary Online Material

Cyclooxygenase-2 /Prostaglandin E2 pathway facilitates Infectious Bronchitis Virus-Induced Necroptosis in Chicken Macrophages, a Caspase-Independent Cell Death

Motamed Elsayed Mahmoud [1,2], Dylan Tingley[1], Akeel Faizal[1], Awais Ghaffar[1], Doaa Salman[1], Ishara M. Isham [1], Mohamed Faizal Abdul-Careem [1,*]

[1] Faculty of Veterinary Medicine, University of Calgary, 3330 Hospital Drive NW, Calgary, AB, T2N 4N1, Canada

[1] Department of Animal Husbandry, Faculty of Veterinary Medicine, Sohag University, Sohag 84524, Egypt.

*Correspondence: Dr. Mohamed Faizal Abdul-Careem, Email: faizal.abdulcareem@ucalgary.ca; Phone +1-403-220-4462

Keywords: Infectious Bronchitis Virus; COX-2/PGE2 pathway; inflammatory cell death; apoptosis; necroptosis; NLRP3 Inflammasome; RIPK1.

Methods

Cytotoxicity assay by MTT

The cytotoxicity assay was conducted following the instructions provided by the manufacturer of the MTT assay kit (MTT Assay Kit Cell Proliferation, ab211091, Abcam). Macrophages were seeded at 100 µl/well in 96-well plates at a density of $1×10^6$ cells/ml and incubated at 40°C for 24 hours. Subsequently, the culture media was removed, and cells were treated with 50 µl of serum-free media followed by 50 µl of MTT solution, then incubated at 37°C for 3 hours. Then, 150 µl of MTT solvent was added to each well and the plates were shaken at 200 rpm for 15 minutes in darkness. The optical density was measured at 595 nm

using the BioRAD microplate reader (680 XR Microplate Reader, Bio-Rad, USA). The cytotoxicity was calculated as a percentage of control-non-treated cells. Each concentration of drug was tested in 8 technical replicates.

Double immunofluorescence for colocalization of IBV and PGE2

Chicken macrophages were cultured on sterile coverslips in 12-well plates and infected with infectious bronchitis virus (IBV) at the 0.1 MOI. At 24 hours post infection (hpi) , cells were washed, fixed with 4% paraformaldehyde, permeabilized with 0.2% Triton X-100, and blocked with 2.5% Horse serum in PBS. Double immunofluorescence staining was performed using primary antibodies against PGE2 and IBV (N-antigen), followed by Alexa Fluor 488- and Alexa Fluor 594-conjugated secondary antibodies for PGE2 (green) and IBV (red), respectively. Cell walls and nuclei were stained with violet Alexa Fluor 647 and DAPI, respectively. Coverslips were mounted on slides, and images were acquired using a Nikon Spectral Confocal Microscopy System. Colocalization of IBV and PGE2 was quantified by assessing the overlap of red and green fluorescence signals using software analysis tools and calculating colocalization coefficients.

Activation of Caspase-1 and Caspase-9 in Chicken Macrophages

To activate caspase-1, chicken macrophages were treated with lipopolysaccharide (LPS) derived from the outer membrane of Gram-negative bacteria (b:001) for 6 hours and 24 hours [Martinon et al., 2002]. For the induction of DNA damage and subsequent activation of caspase-9, cells were exposed to ultraviolet (UV) radiation using a lamp emitting at 365 nm, with an energy dose of 10 mJ/cm^2 for 10 minutes [Norbury, Zhivotovsky, 2004). Following treatment, cells were harvested at 6 and 24 hours post-exposure for analysis.

Statistical analysis

The data underwent analysis using one-way ANOVA followed by Bonferroni post hoc test for group comparisons. Asterisks denote values with statistical significance at $p < 0.05$.

Results

In our study, we investigated the inhibitory effects of BSP at a non-toxic dose (200 μM) on LPS-mediated inflammatory cytokine production in murine RAW264.7 macrophages [Cui et al., 2020 #1]. Based on the MTT cytotoxicity assay conducted across a range of doses (0, 0.2, 2, 20, 200, 2000 μM, Fig. S1A), we selected a concentration of 20 μM for BSP, ensuring non-cytotoxicity.

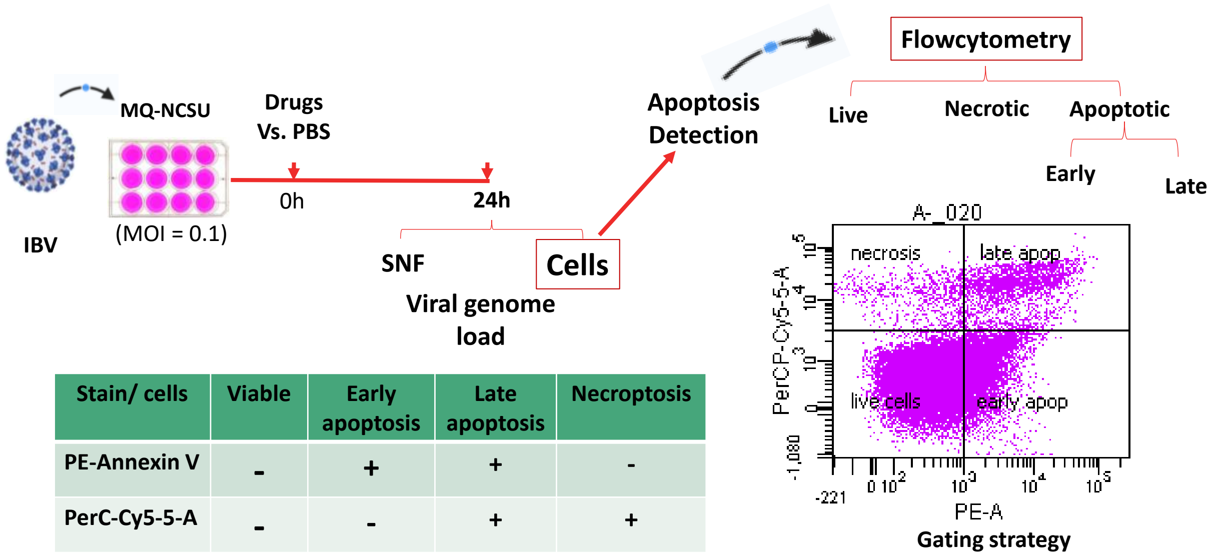

Stain/ cells	Viable	Early apoptosis	Late apoptosis	Necroptosis
PE-Annexin V	-	+	+	-
PerC-Cy5-5-A	-	-	+	+

Figure S1. Schematic representation of gating analysis in flow cytometry. Flow cytometric analysis using the PE-Annexin-V apoptosis detection kit was employed to determine the percentages of viable (bottom left quadrant), early apoptotic (bottom right quadrant), late apoptotic (top right quadrant), and necroptotic (top left quadrant) cells. Viable cells were negative for both stains, late apoptotic cells were positive for both stains, while early apoptotic and necroptotic cells were positive for one stain only.

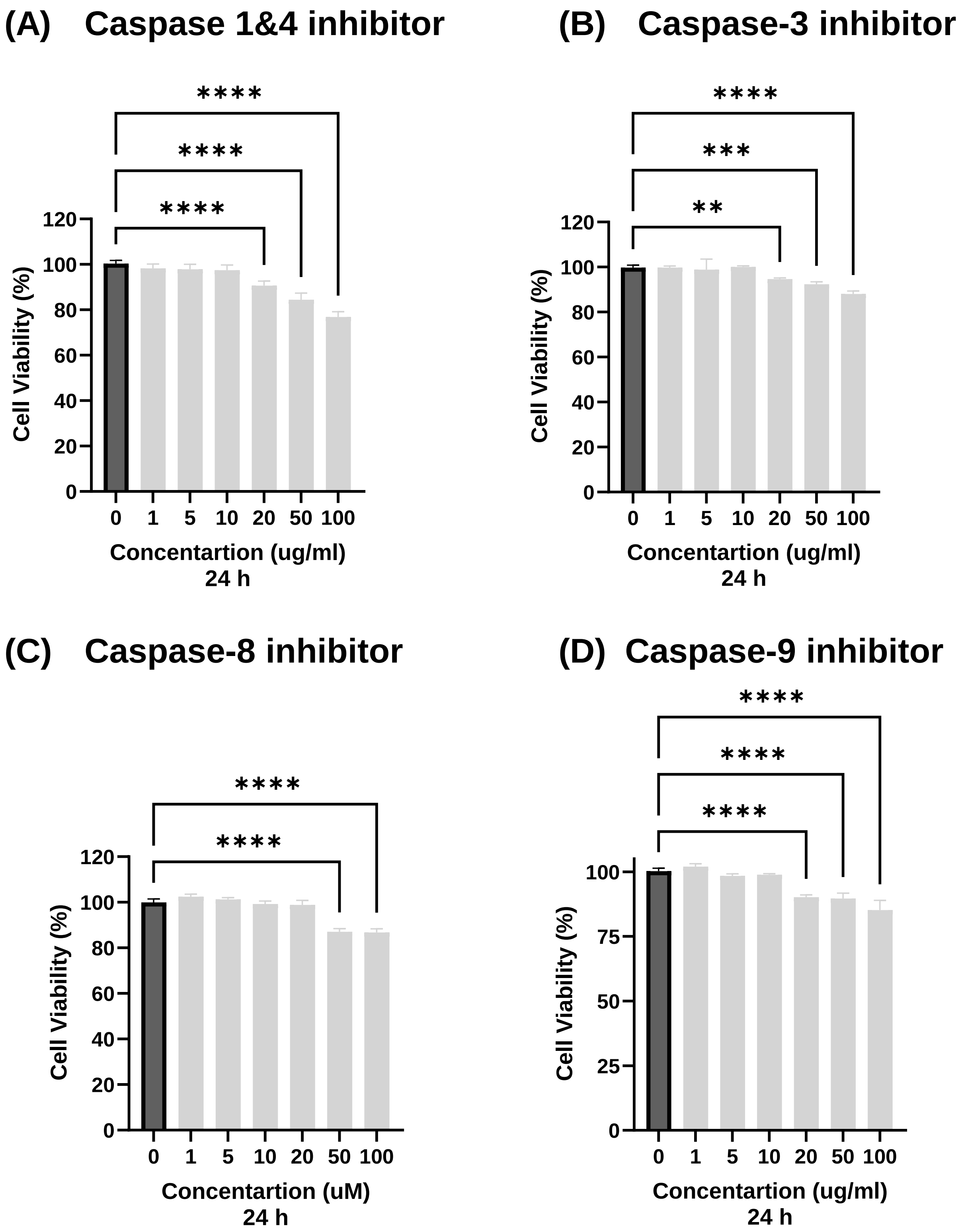
(A) Caspase 1&4 inhibitor
****
****
****
Cell Viability (%)
120
100
80
60
40
20
0
0 1 5 10 20 50 100
Concentartion (ug/ml)
24 h
(B) Caspase-3 inhibitor
****
***
**
Cell Viability (%)
120
100
80
60
40
20
0
0 1 5 10 20 50 100
Concentartion (ug/ml)
24 h
(C) Caspase-8 inhibitor
****
****
Cell Viability (%)
120
100
80
60
40
20
0
0 1 5 10 20 50 100
Concentartion (uM)
24 h
(D) Caspase-9 inhibitor
****
****
****
Cell Viability (%)
100
75
50
25
0
0 1 5 10 20 50 100
Concentartion (ug/ml)
24 h

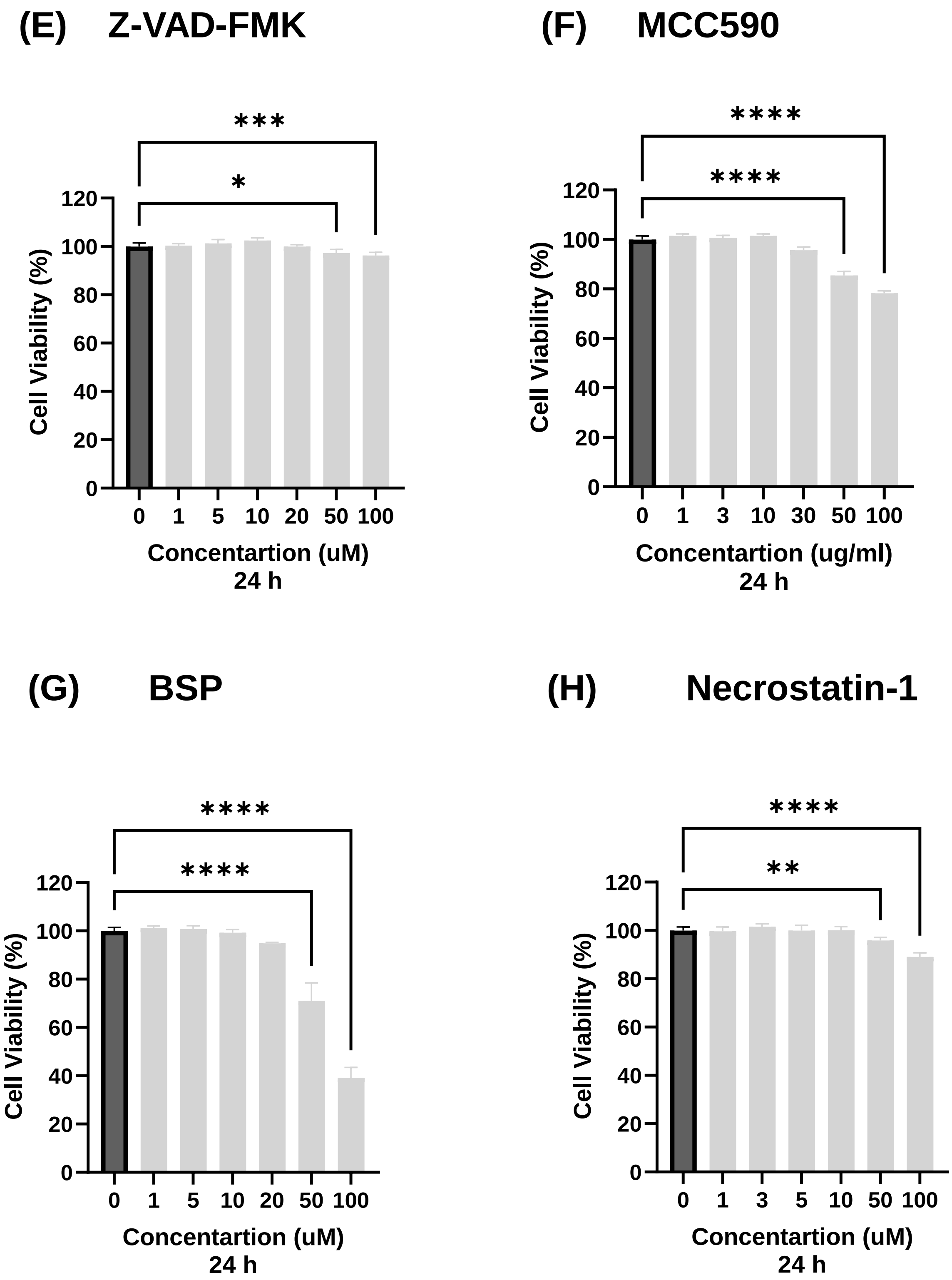

Figure S2. Viability of chicken macrophages treated with various concentrations of drug inhibitors under serum-free conditions. Chicken macrophages were treated for 24 hours with inhibitors targeting caspases (4, 3, 8, 9, and broad-spectrum z-VAD-FMK), prostaglandin

transporters (BSP), the NLRP3 inflammasome (MCC950), and necroptosis (Necrostatin-1). Cell viability was assessed calorimetrically by using the MTT assay in ELISA plate reader. Cells were treated with a concentration gradient of each drug, and each concentration was assayed in 6 technical replicates. Data were statistically analyzed using one-way ANOVA followed by Bonferroni's post-hoc test for multiple comparisons. Values are presented as means ± SD, with comparisons made against control (non-treated) cells. Asterisks indicate statistical significance at (*$p < 0.05$, ** $p < 0.01$, *** $p < 0.001$, **** $p < 0.0001$).

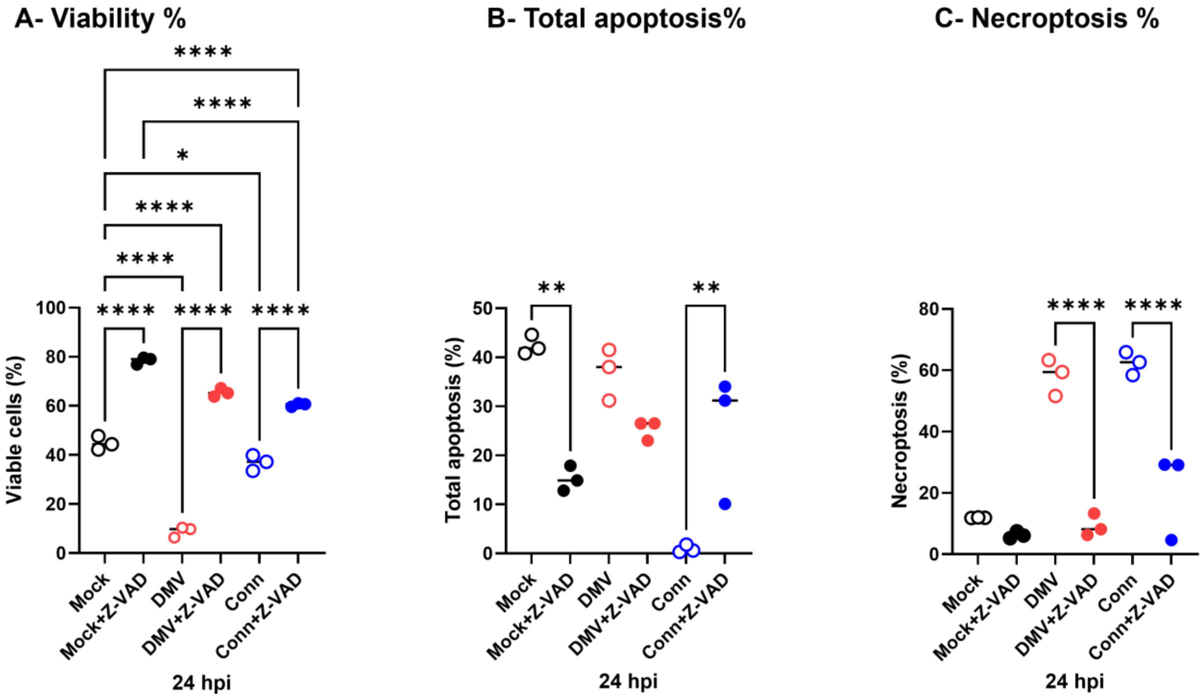

Figure S3. Enhanced necroptosis in chicken macrophages infected with IBV DMV/1639 and IBV Conn A5968 under serum-free conditions. After 1 hour adsorption with IBV, cells were treated with Z-VAD-FMK (25 µM) or PBS for 24 hours. The percentages of viable (A), apoptotic (B), and necroptotic cells (C) were determined through flow cytometric analysis using the PE-Annexin-V apoptosis detection kit. Mean ± SD data were presented and analyzed by one-way ANOVA followed by Bonferroni post hoc test for group comparisons. Data represent the results of two independent experiments (n = 3 per experiment). Values are presented as mean ± SD. Asterisks indicate statistically significant differences (*$p < 0.05$, ** $p < 0.01$, *** $p < 0.001$, **** $p < 0.0001$).

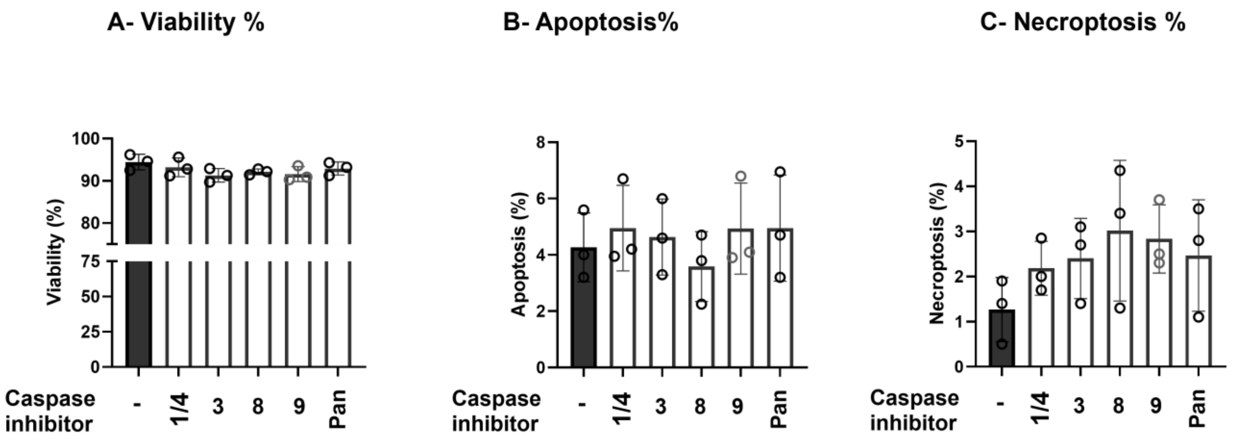

Figure S4. Effects of 24-hour treatment with caspase inhibitors on the viability, apoptosis, and necroptosis of chicken macrophages under 4.5% serum conditions. Chicken macrophages were treated with inhibitors of caspases1/4 (10 µg/ml), caspase 3, (10 µg/ml), caspase 8 (10 µM/ml), caspase 9 (10 µg/ml), or a pan-caspase inhibitor (Z-VAD-FMK, 20 µM), or PBS as a control. The percentages of viable (A), apoptotic (B), and necroptotic cells (C) were determined via flow cytometric analysis using the PE-Annexin-V apoptosis detection kit. Data are presented as mean ± SD and were analyzed by one-way ANOVA followed by Bonferroni post hoc test for group comparisons. Results are representative of one experiment (n = 3).

Total apoptosis

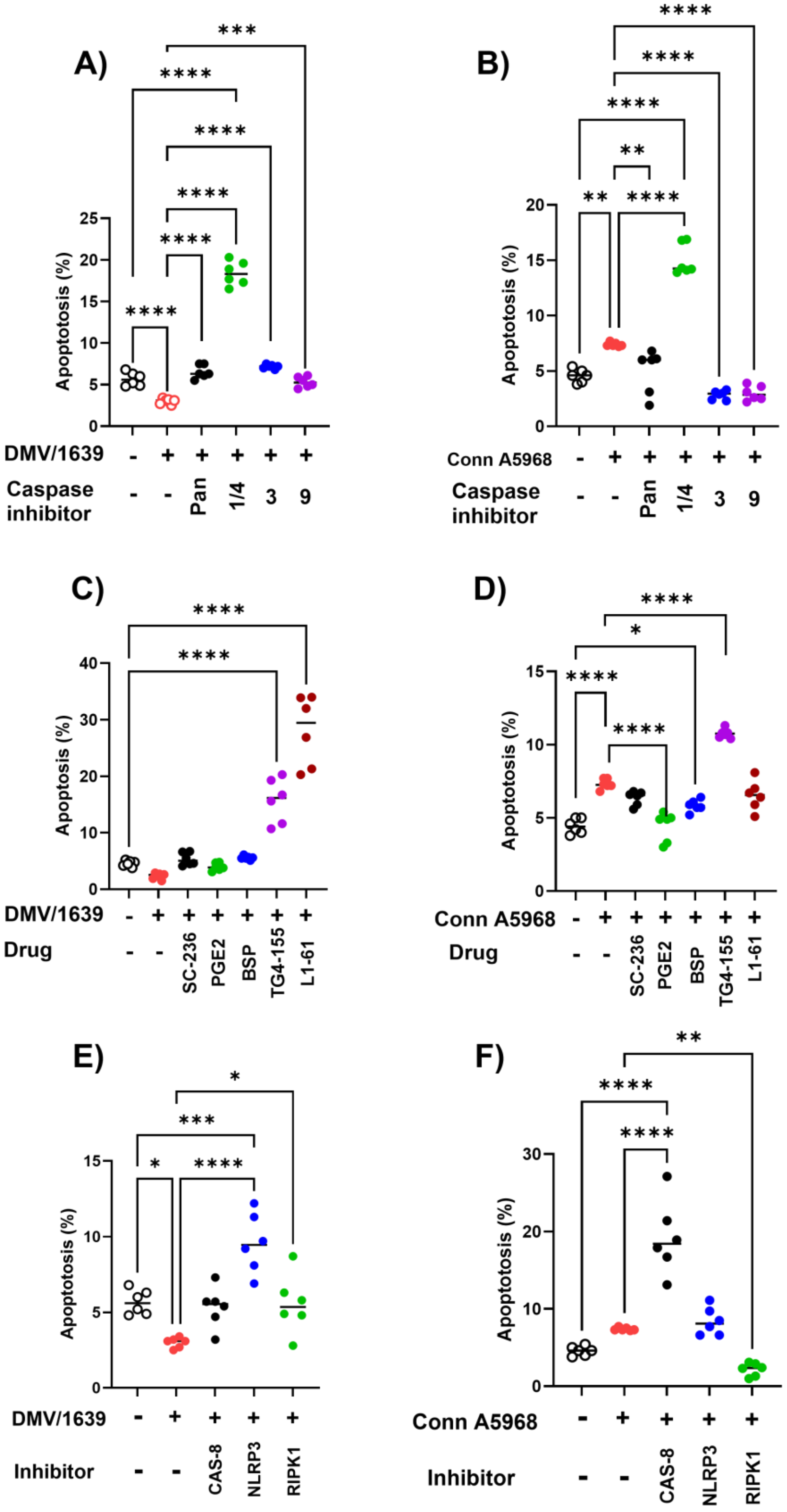

Figure S5. Effects of caspase inhibitors, COX-2/PGE2 pathway modulation, and NLRP3 and RIPK1 inhibitors on apoptosis in chicken macrophages under 4.5% serum conditions. IBV (DMV/1639 or Conn A5968)-infected chicken macrophages were treated with inhibitors targeting caspase-1/4 (10 μg/mL), caspase-3 (10 μg/mL), caspase-8 (10 μM), caspase-9 (10 μg/mL), or a pan-caspase inhibitor (Z-VAD-FMK; 20 μM). Additional treatments included a COX-2 inhibitor (SC-236; 10 μg/mL), exogenous prostaglandin E2 (PGE2; 10 μg/mL), an NLRP3 inflammasome inhibitor (MCC950; 10 μM), a RIPK1 inhibitor (Necrostatin-1; 25 μM), or PBS as a control in culture medium containing 4.5% serum. Apoptosis was assessed by flow cytometry using the PE-Annexin V apoptosis detection kit, and total apoptotic cell percentages were calculated. Data are presented as mean ± SD and analyzed by one-way ANOVA followed by Bonferroni post hoc test. Results are representative of a single experiment (n = 3). Asterisks indicate statistically significant differences (*$p < 0.05$, ** $p < 0.01$, *** $p < 0.001$, **** $p < 0.0001$).

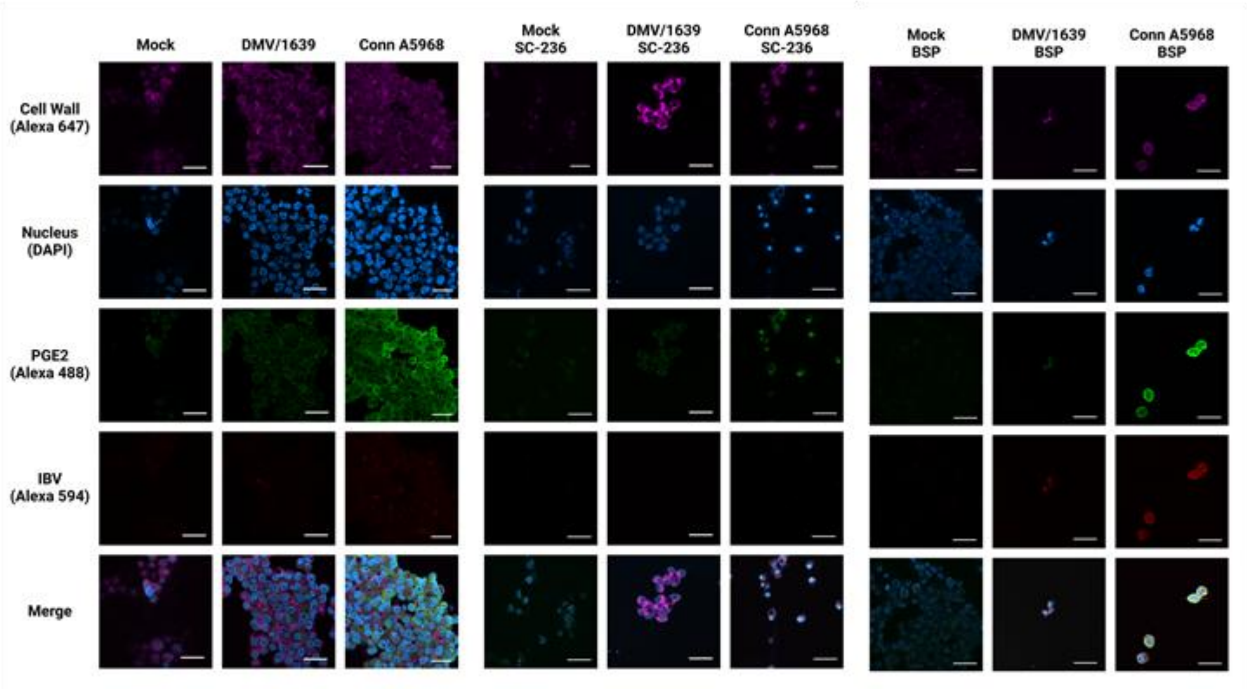

Figure S6. Double immunofluorescence staining of chicken macrophages infected with IBV and stained for PGE2. Chicken macrophages were cultured on sterile coverslips and infected with infectious bronchitis virus (IBV) at 0.1 MOI. At 24 hpi, Immunostaining was performed using primary antibodies against PGE2 and IBV, followed by Alexa Fluor 488- and Alexa Fluor 594-conjugated secondary antibodies for PGE2 (green) and IBV (red), respectively. Cell walls and nuclei were stained with violet Alexa Fluor 647 and DAPI, respectively. Colocalization of IBV and PGE2 was quantified by assessing the overlap of red and green fluorescence signals. The scale bar represents 25 μm.

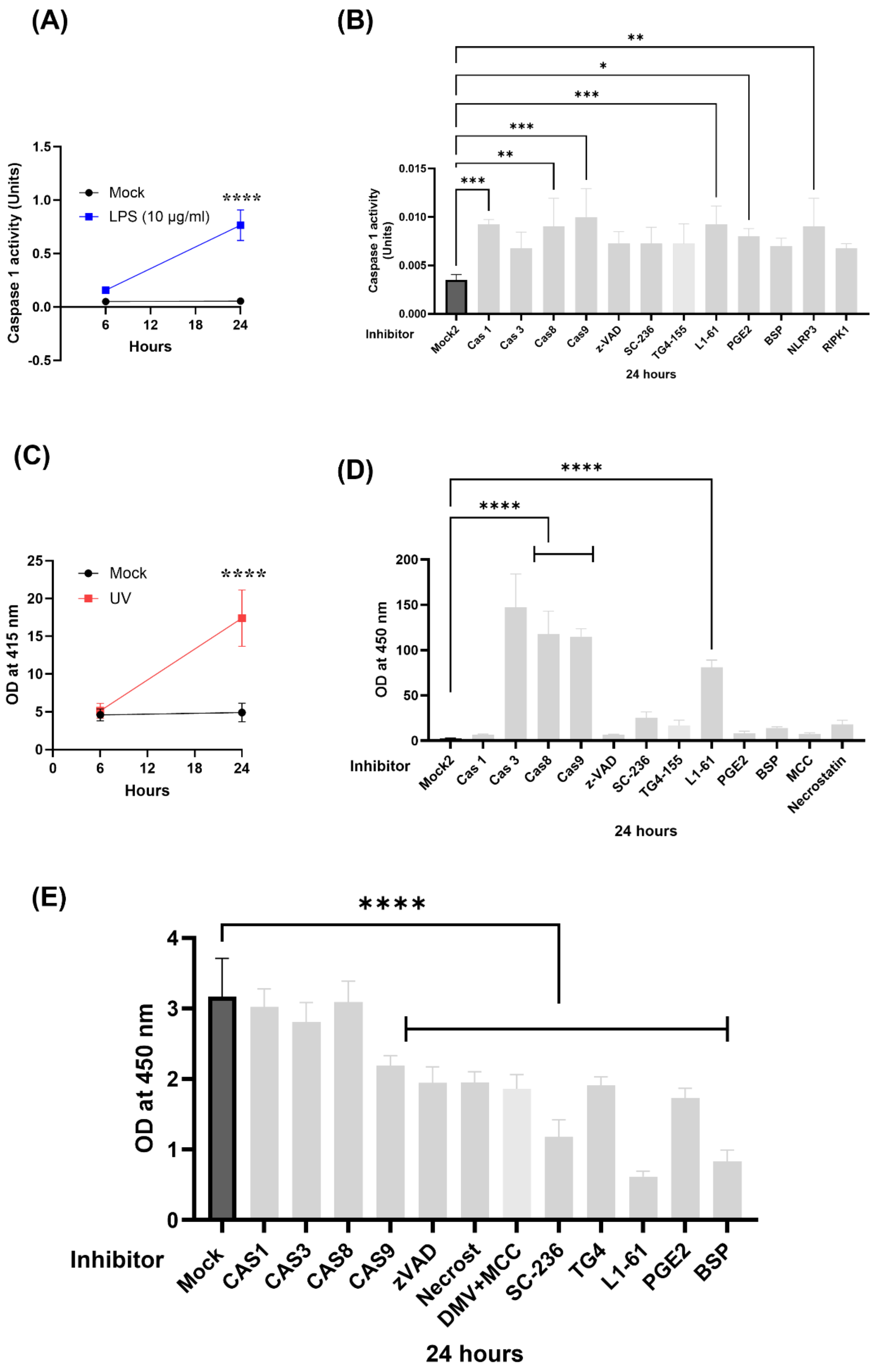

(A)
Caspase 1 activity (Units)
1.5
1.0
0.5
0.0
-0.5
Mock
LPS (10 µg/ml)
****
6
12
18
24
Hours
(B)
**
*
***
***
**
***
0.015
0.010
0.005
0.000
Caspase 1 activity (Units)
Inhibitor
Mock2
Cas 1
Cas 3
Cas8
Cas9
z-VAD
SC-236
TG4-155
L1-61
PGE2
BSP
NLRP3
RIPK1
24 hours
(C)
OD at 415 nm
25
20
15
10
5
0
Mock
UV
****
0
6
12
18
24
Hours
(D)
****
****
OD at 450 nm
200
150
100
50
0
Inhibitor
Mock2
Cas 1
Cas 3
Cas8
Cas9
z-VAD
SC-236
TG4-155
L1-61
PGE2
BSP
MCC
Necrostatin
24 hours
(E)
****
OD at 450 nm
4
3
2
1
0
Inhibitor
Mock
CAS1
CAS3
CAS8
CAS9
zVAD
Necrost
DMV+MCC
SC-236
TG4
L1-61
PGE2
BSP
24 hours

Figure S7. Caspase-1 Activity, Caspase-9 Assay and NF-κB Activation, in Chicken Macrophages Treated with Inhibitors Under Serum-Free Conditions. Chicken macrophages were treated for 24 hours with inhibitors targeting caspases (Caspase-1/4, -3 (10 μM), -8, -9, and broad-spectrum caspases (Z-VAD-FMK; 25 μM)), the NLRP3 inflammasome (MCC950; 10 μM), RIPK1 (Necrostatin-1; 25 μM) (A, C), or COX-2 inhibitor (SC-236; 10 μg/ml), exogenous PGE2 (10 μg/ml), the PGE2 transporter inhibitor (BSP; 25 μM), and EP receptor antagonists (EP2: TG4-155; 4 μM and EP4: L1-61; 8 μM) for 24 hours in serum-free culture media. For caspase-1 induction, cells were treated with lipopolysaccharide (LPS, 10 μg/ml) (**A**). For caspase-9 induction (**C**), cells were exposed to UV radiation for 10 minutes and incubated for 6 or 24 hours. **E)** NF-κB activation was quantified calorimetrically using the IKK-α (Phospho-Ser176)/IKK-β (Phospho-Ser177) Cell-Based ELISA assay. **A,B)** Caspase-1 activity was measured using the Caspase-1 Colorimetric Assay, and **C,D)** Caspase-9 activity was assessed using the Caspase-9 Colorimetric Assay. Absorbance values were measured with an ELISA plate reader. Each treatment was performed in four technical replicates. Statistical analysis was conducted using one-way ANOVA followed by Bonferroni's post-hoc test for multiple comparisons. Data are presented as mean ± SD, with comparisons made against untreated control cells. Asterisks denote statistically significant differences (*$p < 0.05$, ** $p < 0.01$, *** $p < 0.001$, **** $p < 0.0001$).

References:

Cui, F., Sequeira, S. B., Huang, Z., Shang, G., Cui, Q., & Yang, X. (2020). Bromosulfophthalein suppresses inflammatory effects in lipopolysaccharide-stimulated RAW264.7 macrophages. *Immunopharmacology and immunotoxicology*, *42*(5), 456–463. https://doi.org/10.1080/08923973.2020.1808985

Jin, Y., et al. (2022). "Novel role for caspase 1 inhibitor VX765 in suppressing NLRP3 inflammasome assembly and atherosclerosis via promoting mitophagy and efferocytosis." Cell Death & Disease **13**(5): 512.

Li X, Yao X, Zhu Y, et al. The Caspase Inhibitor Z-VAD-FMK Alleviates Endotoxic Shock via Inducing Macrophages Necroptosis and Promoting MDSCs-Mediated Inhibition of Macrophages Activation. Frontiers in Immunology. 2019;10:1824. DOI: 10.3389/fimmu.2019.01824. PMID: 31428103; PMCID: PMC6687755.

Martinon, F., Burns, K., & Tschopp, J. (2002). The inflammasome: a molecular platform triggering activation of inflammatory caspases and processing of proIL-beta. *Molecular Cell*, 10(2), 417-426.

Norbury, C. J., & Zhivotovsky, B. (2004). DNA damage-induced apoptosis. *Oncogene*, 23(16), 2797-2808.
